# Mother-Specific Signature in the Maternal Transcriptome Composition of Mature, Unfertilized *Zebrafish* Eggs

**DOI:** 10.1371/journal.pone.0147151

**Published:** 2016-01-22

**Authors:** Han Rauwerda, Paul Wackers, Johanna F. B. Pagano, Mark de Jong, Wim Ensink, Rob Dekker, Ulrike Nehrdich, Herman P. Spaink, Martijs Jonker, Timo M. Breit

**Affiliations:** 1 RNA Biology & Applied Bioinformatics research group, Swammerdam Institute for Life Sciences, Faculty of Science, University of Amsterdam, Amsterdam, the Netherlands; 2 Institute of Biology Leiden, Faculty of Science, Leiden University, Leiden, the Netherlands; National University of Singapore, SINGAPORE

## Abstract

Maternal mRNA present in mature oocytes plays an important role in the proper development of the early embryo. As the composition of the maternal transcriptome in general has been studied with pooled mature eggs, potential differences between individual eggs are unknown. Here we present a transcriptome study on individual *zebrafish* eggs from clutches of five mothers in which we focus on the differences in maternal mRNA abundance per gene between and within clutches. To minimize technical interference, we used mature, unfertilized eggs from siblings. About half of the number of analyzed genes was found to be expressed as maternal RNA. The expressed and non-expressed genes showed that maternal mRNA accumulation is a non-random process, as it is related to specific biological pathways and processes relevant in early embryogenesis. Moreover, it turned out that overall the composition of the maternal transcriptome is tightly regulated as about half of the expressed genes display a less than twofold expression range between the observed minimum and maximum expression values of a gene in the experiment. Even more, the maximum gene-expression difference within clutches is for 88% of the expressed genes lower than twofold. This means that expression differences observed in maternally expressed genes are primarily caused by differences between mothers, with only limited variability between eggs from the same mother. This was underlined by the fact that 99% of the expressed genes were found to be differentially expressed between any of the mothers in an ANOVA test. Furthermore, linking chromosome location, transcription factor binding sites, and miRNA target sites of the genes in clusters of distinct and unique mother-specific gene-expression, suggest biological relevance of the mother-specific signatures in the maternal transcriptome composition. Altogether, the maternal transcriptome composition of mature *zebrafish* oocytes seems to be tightly regulated with a distinct mother-specific signature.

## Introduction

Maternal RNA is defined as those transcripts present in a mature oocyte or (un)fertilized egg before the initiation of zygotic gene expression [1,2]. Their significance is reflected in the enormous amounts, up to several ug total RNA per egg in oviparous species [3]. Oogenesis is a complex process in which, during the very early stages DNA replication takes place [4]. After that, but still in the Prophase I of the meiosis chromosomes unpair and the developing oocyte starts a long period of cytoplasmic growth. In this lengthy developmental phase the maternal RNA molecules are either produced by the oocyte or deposited by surrounding cells into the oocyte during oogenesis [5,6]. Spatiotemporal localization of maternal RNAs is at least for germ plasm RNAs highly dynamic [7]. During oogenesis and early embryogenesis mRNA is actively deadenylated and polyadenylated [8]. In the oocyte a large number of RNA molecules is stored in the cytoplasm with short poly(A) tails that can acquire translational competence after cytoplasmic polyadenylation during oocyte maturation and embryogenesis [9]. Only after fertilization the second phase of the meiosis is resumed and the second polar body is produced. Maternal mRNAs provide a means of protein production without transcription in early embryogenesis [2,10,11]. Thus, besides functions in the developing oocyte, these maternal gene products play an important role in the proper development of the early embryo, such as the start of the zygotic transcription [1,12]. During the maternal-to-zygotic transition (MZT) maternal mRNAs are cleared from the embryo in a highly regulated fashion [13]. Fish maternal mRNA is intensively studied because *zebrafish* (*Danio rerio*) is an increasingly popular model organism [14] and maternal RNAs are considered important determinants of egg quality in farmed fish [15].

Over the years, many studies have been carried out to elucidate the role of maternal mRNA in oogenesis and early embryogenesis including whole-genome transcriptomics experiments. This resulted in many new insights on the functioning of maternal mRNAs. However, as most studies, including those on *zebrafish* [16–19], *C*. *elegans* [20], *T*. *castaneum* [21], *D*. *melanogaster* [22] and *human* [23], have been performed using pools of oocytes, eggs or embryos, little is known about the maternal mRNA differences between individual oocytes. The few studies using single cell transcriptome analyses [24,25] do not specifically address the transcriptome variation between oocytes. Another complicating factor in maternal RNA experimentation is the fact that in the oocyte, many maternal RNAs have no or short poly(A) tails plus that mRNAs may be specifically polyadenylated or deadenylated during early embryogenesis [9,26,8]. Therefore the often used poly(A)-based selection or amplification protocols will likely result in a biased outcome [13].

Given the important role of maternal mRNA, the constitution of the maternal transcriptome of an individual egg will have a profound impact on the eventual individual [27–30]. This, in fact, might be an epigenetics means to transmit (environmental) information from the mother to the offspring [31,32]. It has been shown that the epigenetic factor DNA methylation plays a role in the transmission of environmental stimuli into the embryo development [33–36]. An approach to investigate the role of maternal mRNA in transmission of such epigenetic information would be to analyze the differences in the maternal transcriptomes between sibling oocytes in one clutch and clutches from different mothers.

**Fig 1:**
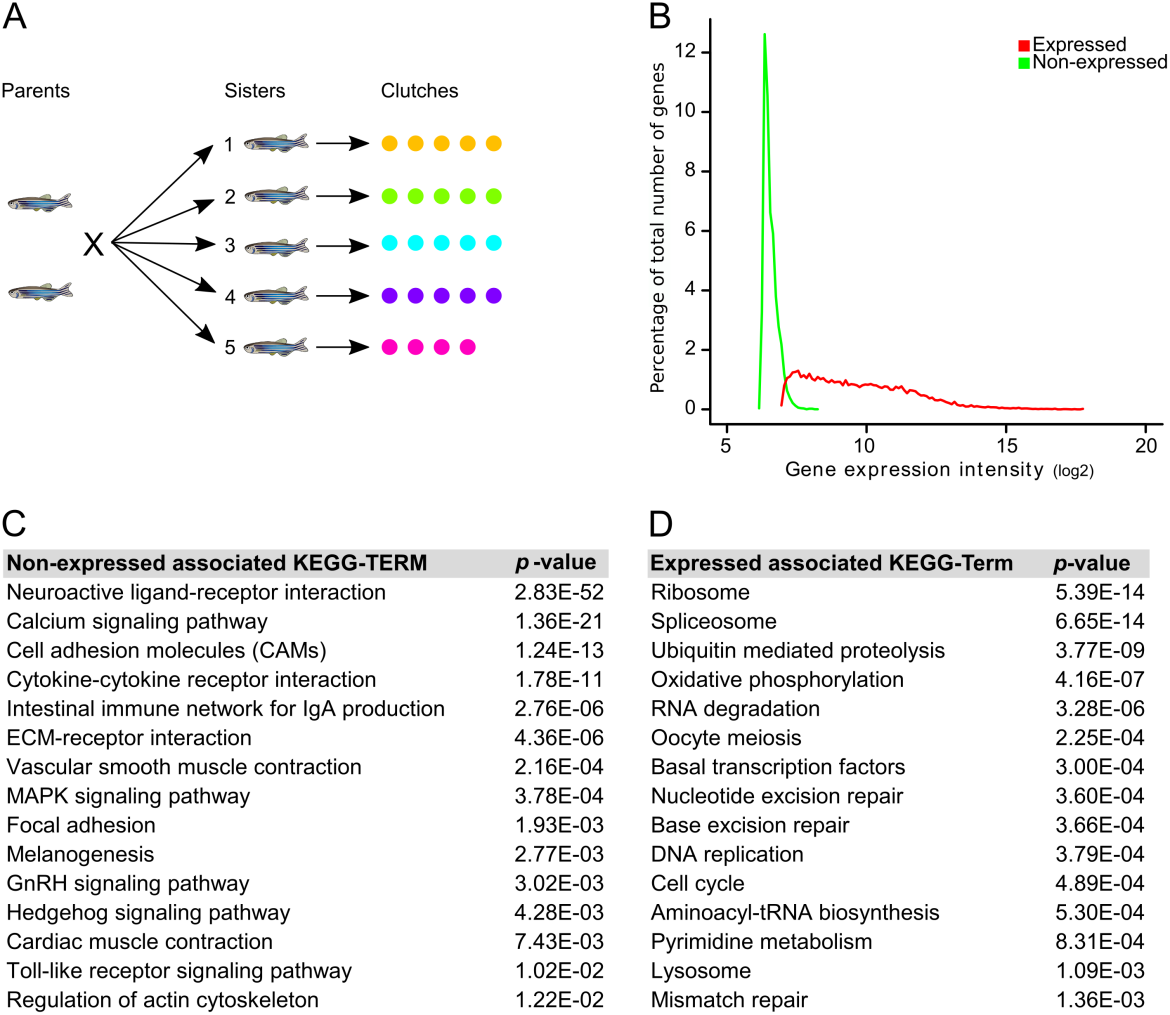
Maternal gene expression in *zebrafish* eggs. (A) Experiment design: the transcriptomes of 24 individual *zebrafish* eggs was determined in five clutches. (B) Gene-expression distribution of the 11,118 expressed (red) and 11,353 non-expressed (green) unique Ensembl-defined genes. (C) Top 15 over-represented KEGG pathways associated with the maternally non-expressed genes. (D) Top 15 over-represented KEGG pathways associated with the maternally-expressed genes.

Accordingly, we performed an experiment using four to five eggs per clutch from five different mothers, using our single-egg transcriptome-analysis technique [37] that allows us to detect inter-individual differences (Fig 1A). To avoid the maternal mRNA short poly(A) tail complication, we depleted the maternal RNA samples for rRNA and subsequently polyadenylated the remaining RNA. To limit the influence of genetic differences, we used sibling mothers. The transcriptome analysis showed that there is surprisingly limited differential gene expression between individual eggs, especially within one clutch. This indicates the existence of a tightly regulated gene-expression program in oogenesis. Even though these differences are relatively small, over 99% of expressed genes show in at least one between-clutch comparison a statistically different gene expression. This means that there seems to be a mother-specific influence in the maternal transcriptome of these mature eggs. Importantly, the combined differential gene-expression patterns exclude the possibility that these differences originate from eggs that are in a different state of maturation, activation, meiosis, or decay. Hence, we conclude that each clutch has a highly similar maternal transcriptome yet with a noticeable signature of the mother. These differences seem to be controlled by direct gene regulation as they involve gene sets with a non-random chromosome distribution, transcription factor binding sites in their promotor regions, or differential miRNA targets in their 3’ UTRs.

## Material and Methods

### *Zebrafish* eggs

*Zebrafish* were handled in compliance with the local animal welfare regulations and maintained according to standard protocols (http://ZFIN.org). Mature, unfertilized eggs were collected from 5 different animals (sisters) of genotype wt ABTL by a squeezing procedure as described in http://zfin.org/zf_info/zfbook/chapt2/2.8.html#4. In short, a female, looking large and fat in the belly and a male were placed in one tank separated by a transparent, but watertight division the day prior to the squeezing procedure. Approximately 1 hour after the start of the light period, the division was removed and 3 minutes later and before any egg laying or sperm deposition took place, the female was removed from the tank and anesthetized briefly in another tank with egg water containing 0.02% buffered ethyl 3-aminobenzoate methanesulfonate (Tricaine, Sigma-Aldrich). Subsequently, the eggs were obtained by gently squeezing the female. Finally, some egg water was added to the clutch to facilitate the picking of individual eggs. During a period of 1 to maximally 15 minutes after expulsion of the clutch, the mature eggs were one after another, individually put into Eppendorf tubes, flash-frozen in liquid nitrogen, and stored at -80°C. The local animal welfare committee (DEC) of the University of Leiden, the Netherlands specifically approved this study. All protocols adhered to the international guidelines specified by the EU Animal Protection Directive 86/609/EEC. From each female, five eggs were taken from which RNA was extracted.

### RNA extraction & microarrays

RNA was isolated from single eggs as described previously (de Jong et al., 2010). RNA concentrations were measured on a NanoDrop ND-2000 (Thermo Scientific), S1 File, and RNA integrity was assessed on a 2200 TapeStation system using RNA ScreenTapes (Agilent Technologies). Total RNA was polyadenylated using the Poly(A) Polymerase Tailing Kit (Epicentre). Prior to amplification and labeling, polyadenylated total RNA was depleted for ribosomal RNA (rRNA) using a set of biotinylated single-stranded DNA oligonucleotides designed to hybridize to Danio rerio 18S and 28S rRNA (S2 File). A detailed ribo-depletion protocol can be found in the S3 File. Polyadenylated ribo-depleted RNA was amplified and labeled using amino-allyl-CTP (TriLink Biotechnologies) in a modified version of a standard T7 RNA polymerase-based in vitro transcription protocol [38]. Amplified RNA was purified using the E.Z.N.A. MicroElute RNA Clean Up Kit (Omega Bio-Tek), quantified using a NanoDrop ND-2000 and its quality was checked on RNA ScreenTapes (Agilent Technologies). A reference sample—mentioned here for completeness, since in the analysis a one dye approach was applied—was compiled from a pool of eggs.

Test and reference samples were labeled with Cy3 and Cy5, respectively, by incubating 5 μg of amino-allyl labeled amplified RNA in 50 mM carbonate buffer (pH 8.5) with Cy3 or Cy5 mono-reactive dyes (GE Healthcare) for 1 hour at room temperature. Reactions were quenched with hydroxylamine (Sigma-Aldrich), purified with the E.Z.N.A. MicroElute RNA Clean Up Kit and the yield and CyDye incorporation were measured on a NanoDrop ND-2000. Finally, 4.4 ug test and 2.2 ug reference labeled amplified RNA was combined and hybridized to custom-designed D. rerio 4x180k SurePrint G3 microarrays (Agilent Technologies, GEO accession GPL15180) for 17 hours at 65°C. In order to avoid confounding of clutch and hybridization chamber samples were randomized. The microarrays were scanned in an ozone-free room with a DNA microarray scanner G2565CA (Agilent Technologies). Feature extraction was performed with Agilent Feature Extraction software.

In short, these microarrays were designed by applying the method described in [39], albeit with a few modifications: for each transcript only one probe was designed at the 3’ end of the transcript. The transcripts were based on Ensembl 57, Vega37, Unigene117 and Refseq39. This set comprises of 88,754 transcripts and 24 negative control probes and is a subset of the microarray design described in GEO GPL 10042 (http://www.ncbi.nlm.nih.gov/geo/query/acc.cgi?acc=GPL10042). Next to this set the array also contains 44k probes from older, redundant array designs, which were not used in this experiment.

### Data preprocessing & normalization

The quality of the microarray data was assessed via multiple quality-control checks, i.e. visual inspection of the scans, testing against criteria for foreground and background signals, testing for consistent performance of the labeling dyes, checking for spatial effects through pseudo-color plots, and inspection of pre- and post-normalized data with box plots, ratio-intensity (RI) plots and PCA plots. All microarrays passed the minimum quality criteria and were used in the analyses. Handling, analysis and visualization of all data was performed in R (http://cran.r-project.org/) using the Bioconductor packages affy, limma and maanova [40]. At this stage we removed two genes (rdh14b and zgc:63480) from the dataset, due to the fact that they each had an extreme high expression value in one of the samples, whereas the expression was consistently absent in all other samples, which resulted in a unrealistic fold changes of over 3,000 times. We also removed 194 genes which microarray signals could not be normalized as they apparently already produce polyadenylated maternal RNA by themselves (S1 Fig, S4 File). Log2 transformed Cy3 data was normalized between microarrays by quantile normalization from the Robust Multi-microarray Average (RMA) function in the R Bioconductor affy package. Raw and processed data are deposited in the Gene Expression Omnibus accession GSE72839 (http://www.ncbi.nlm.nih.gov/geo/query/acc.cgi?token=gnahakmgtjurryh&acc=GSE72839).

### Data analysis

In order to distinguish expressed genes from non-expressed genes, we developed a sequential procedure to: determine which probes are expressed per sample/microarray; how these expressed probes translate to expressed transcripts in the whole experiment; and finally how these expressed transcripts translate to expressed genes in this study. Starting from the raw data and the assumption that high-variant signals are indicative for expressed genes, we determined for each probe the log variance of the log intensity over the entire experiment of the Cy3 channel. In the resulting bimodal distribution (S2A Fig) the log variance between the low- and the high-variance peak was determined at -3.74 with the lowest number of probes below which probes were labeled ‘low-variance probes’ (58,212) and above which probes were labeled ‘high-variance probes’ (30,512). Using Bayes’ theorem a per-microarray per-probe conditional probability was calculated, i.e. that the probe given its intensity value belongs to the high-variance distribution. Here the per-microarray threshold to label a probe as expressed was set at a likelihood of larger than 0.95. With this threshold, interpreted as the intensity above which we accept that a probe is reliably detecting the associated gene products in a specific sample, we are stringently avoiding false positives. To call a transcript “expressed somewhere in the experiment”, we applied a second requirement in that a specific probe must be assigned to the expressed category on at least four microarrays; finally Ensembl transcripts identifiers were linked to their Ensembl gene identifiers where a gene was labeled “expressed” when at least one of its containing transcripts was called “expressed somewhere in the experiment”. In those cases where more transcripts link to one gene, the signal intensity of that gene was determined by the probe with the highest average signal intensity over all samples.

Of all expressed genes the signal intensity range per clutch was determined. If one or more clutches were identified without any overlap with another cluster, these clusters were labeled as clusters with an absolute distinct mother-specific gene-expression level. Hence per gene no to five absolute distinct mother-specific gene-expression levels can be identified. This procedure was carried out by an R script.

The variability of the maternal transcriptome was also assessed by the calculation in R per gene of the maximum fold changes (mFCs) in gene expression between any of the 24 eggs in the study. The mFC within clutches was calculated as the largest of the differences of the highest log intensity in any clutch and the lowest log intensity in the same clutch.

The analysis of variance was carried out with the R Bioconductor maanova package by applying an F-test on the normalized data that was fitted to linear model with ‘clutch’ as model factor. A correction for multiple testing was applied using the ‘jsFDR’ option from the R Bioconductor qvalue package at a false discovery rate of less than 5%.

Comparison of the results in this study with the study of Aanes et al. [16] was done by a direct comparison of the counts of Ensembl genes. Because of annotation differences not all Ensembl genes in the Aanes defined clusters (‘Degradation 1’, ‘Degradation 2’, ‘pre-MBT 1’, ‘pre-MBT 2’, ‘MBT’, ‘Post-MBT’ and ‘Maternal-zygotic’) were present in our set. However, since this difference was very small (2%), we did not perform a mapping of our probes to these missing identifiers. Percentages of overlap were calculated as the ratio of number of genes in our set and the number of genes given in the study of Aanes et al.

Overrepresentation analyses were carried out using the Functional Annotation Tool of the DAVID Bioinformatics Resources version 6.7 [41] using Ensembl gene identifiers and choosing the appropriate background (cf. Results and Discussion) in each comparison (e.g. choosing all on the microarray represented Ensembl genes as background when probing overrepresentation of expressed genes). A Fisher Exact statistics was calculated while a pathway or GO category only was reported if minimally 2 genes in a pathway were identified and the minimum probability of the category was equal or smaller than 0.1.

Hexamer motifs, poly(A) sites (PASs) and Pumilio Binding Elements (PBEs) in the 3’UTRs were determined using the AME tool of the MEME tool suite, version 4.10.1 [42] using the discriminative mode in which the 3’ UTRs of all sequences that were part of the comparison were taken as control sequences and only looking for motifs on the same strand. A Wilcoxon rank-sum test was used, as sequence scoring method an average odds score was used and p-values were adjusted for the effect of multiple testing in a motif set by a Bonferroni procedure. The sequence motifs were retrieved from [43–45]. 3’UTR sequences were retrieved from Ensembl Biomart using Zv9 (Ensembl release 79), as were the chromosome locations of the genes.

The visualization of genes on the assembly was done using the Ensembl website (http://www.ensembl.org/Danio_rerio/Location/Genome, Zv10) by using the ‘View karyotype’ link. Clustering the chromosome distribution of gene sets was performed with a matrix containing the gene sets (rows) and chromosomes (columns) in which the values represent the fraction of that particular gene set represented on that particular chromosome. A hierarchical clustering was performed using Euclidean distance with Ward’s method for linkage.

Analysis of overrepresented transcription-factor binding sites (TFBS) in the upstream region of the genes was performed with the F-MATCH tool of Transfac Professional [46] while choosing an appropriate background (cf. Results and Discussion) consisting of Ensembl genes that are represented on the microarray. The default setting of F-Match were used.

The Ensembl genes that have putative targets of miRNAs in their 3’ UTRs were obtained from TargetScanFish, release 6.2 [47]. The probability of a found overlap of a gene set containing the targets for a specific miRNA in a set of interest was calculated using a hypergeometric test in R by using an appropriate background (cf. Results and Discussion).

## Results and Discussion

### Experiment description

Little is known about the differences in the maternal transcriptomes between individual *Zebrafish* eggs, as to our knowledge in all published studies on transcriptome dynamics in early embryogenesis eggs are pooled for analysis [16–19,48]. Here, we aim to gain insight into the composition of maternal RNA by studying the transcriptome differences between individual mature, unfertilized eggs. For this, we analyzed, by microarray technology, up to five individual eggs from clutches from five different mothers, in total 24 eggs. To minimize the differences caused by genetic background, we used clutches from siblings (Fig 1A). Although mature eggs, even activated, are supposed to be transcriptionally quiescent, we followed a strict protocol in harvesting these mature eggs to avoid changes due to the complete squeezing procedure that is commonly used to obtain a population of synchronized mature eggs.

We anticipated quite some difference in the maternal transcriptomes, as for instance, we previously observed considerable differences in the RNA content of individual eggs [37]. In this study again, there was substantial difference in total RNA isolation yield between eggs, ranging from 80 ng to 474 ng, sd ~ 93 ng (S1 File). However, from a one-way ANOVA using clutch and yield as independent variable and dependent variable, we conclude that the clutches on average might have the same yield (P value = 0.2, with an F-value of 1.6). As we cannot estimate the contribution of the RNA-isolation protocol to the observed differences in yield, it is difficult to conclude what the quantitative RNA differences are between individual eggs. Moreover, if these differences exist, it is unclear whether these are caused by arbitrary gene expression and degradation during oogenesis or reflect a functional different constitution of an egg. Hence, in this study we only analyzed relative mRNA expression levels of genes.

### Maternally-expressed genes

A first step towards gaining insight into the *zebrafish* oocyte transcriptome is to determine which genes are expressed in mature eggs. Given the well-known microarray probe-affinity issues [49,50], as well as the presence of background noise, defining expressed versus non-expressed transcripts is not a trivial task. We argued that both technical and biological causes result in the fact that expressed transcripts exhibit more variation than non-expressed transcripts in raw data. The distribution plot of gene-expression variance (S2A Fig) showed a distinct bimodal distribution of low and high variation, which supports our assumption. The associated average gene-expression profiles exhibited an expected difference in that transcripts with low variation also displayed low gene-expression intensities and reversely, transcripts with high variation displayed high gene-expression intensities (S2B Fig). Employing this approach (cf. Materials and Methods), we could identify 28.276 (32%) transcripts that are expressed in at least four eggs (S2C and S2D Fig), which translates into 11,118 (49%) maternally-expressed unique Ensembl-defined genes and 11,353 non-expressed unique Ensembl-defined genes (Fig 1B and S5 File).

This is in line with the findings of Aanes *et al*. [16] in that the number of expressed genes is similar to their 11,851 Ensembl-defined expressed genes. In this study 6 clusters of genes are described that show a typical behavior in the developmental period from egg to 5.3 hours post fertilization (hpf): ‘Degradation 1’, ‘Degradation2’, ‘Pre-MBT 1’, ‘Pre-MBT 2’, ‘Post-MBT’, ‘Maternal-zygotic’. We found that 88% of the genes in the Aanes-defined clusters that show expression during the oocyte stage (i.e. ‘Degradation 1’, ‘Degradation 2’, ‘Pre-MBT 1’, ‘Pre-MBT 2’ and ‘Maternal-zygotic’, also are expressed genes in our study, while only 9% of the genes in these clusters overlap with the genes that are identified as non-expressed in our study. Also, 81% of the genes in the Aanes-defined clusters with expression at or after the mid-blastula transition (i.e. ‘MBT’ and ‘Post-MBT’) are identified as non-expressed genes in our study (S6 File).

During oogenesis, several miRNAs are implicated in gene-expression regulation [51,52]. Also, in early embryogenesis miRNA play an important role in the clearance of maternal RNAs in the developing zygote [53–55]. Hence, we investigated whether the maternally-expressed genes contained specific miRNA target sites. It indeed turned out that genes containing a miRNA target site were, tested against a background of all available genes on the microarray, overrepresented in the maternally-expressed genes for most oogenesis and early-embryogenesis implicated miRNAs (S7 File). Noticeably, none of the tested miRNAs showed overrepresentation of its target genes in the non-expressed genes.

Early embryonic development relies on the regulated translation of stored maternal RNAs, which is, at least for a large number of proteins, organized through polyadenylation of the maternal RNA molecules. The polyadenylation mechanism involves recognition by dedicated proteins of specific motifs present in the 3’UTRs of the RNAs [44,45]: AAUAAA and AUUAAA hexamers, U-rich cytoplasmic polyadenylation elements (CPEs, UUUUAU and UUUUAMU), and Pumilio Binding Element (PBE, UGUA(N)AUA). Embryonic CPEs (eCPEs) have been reported for Xenopus [56] and for zebrafish [16] as long U-rich motifs larger than 10 nucleotides. These hexamers, CPEs, PBEs and eCPEs should be enriched in the maternally-expressed genes. Analysis of the 3’UTRs of these genes showed that the hexamer motifs were indeed enriched (Bonferroni corrected p-value of 5.4 E-06 and 2.3 E-03, respectively), as were the CPEs (Bonferroni corrected p-value of 8.1 E-05 and 1.4 E-04). The strongest overrepresentation was found in the eCPEs; we tested motifs consisting of stretches Us with a length of 11 to 20 nucleotides and found Bonferroni corrected p-values of 2.95 E-08 for the 11 mer motif increasing gradually to 9.53 E-06 for the 20 mer motif. The tested PBE motif however did not show enrichment. These findings support our categorization of genes as maternally-expressed.

It is of interest to see whether the expressed and non-expressed gene categories relate to any biological mechanism or pathway. For this we performed over-representation analysis on the gene sets in these categories using all genes that are available on the microarray as background (S8 File). From these analyses it became clear that the gene expression is focused rather than random; the non-expressed genes strongly relate to gene pathways that involve many membrane bound and (neurological) signaling gene products (Fig 1C), which are less likely to be relevant in early embryogenesis, whereas the maternally-expressed genes relate very much to basal cellular processes of early embryogenesis relevant processes (Fig 1D), such as transcription, translation, and DNA repair. This is in line with earlier observations [16,19].

### Limited variability in maternal gene expression within clutches

**Fig 2:**
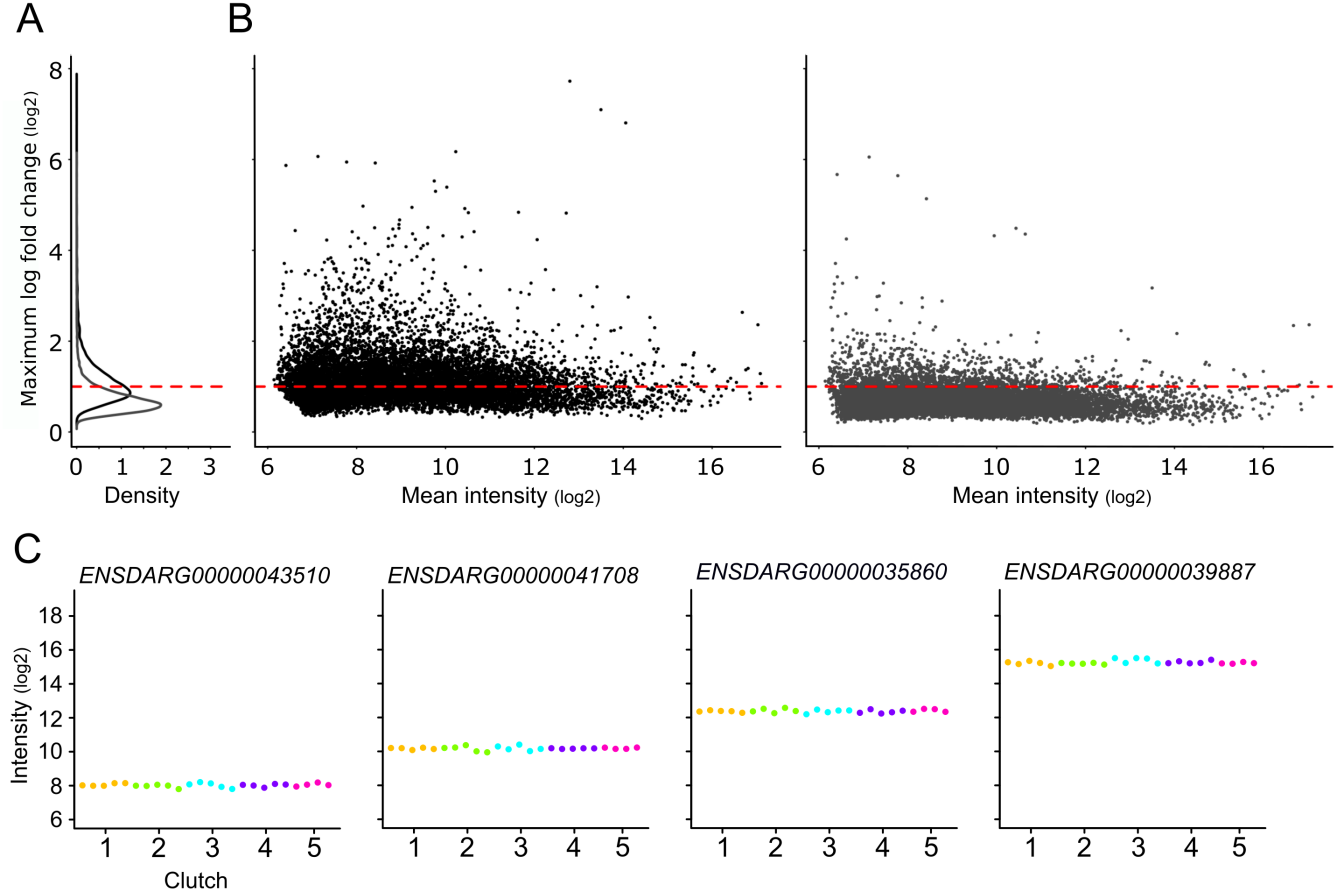
Differential gene expression in the maternal transcriptome. Variability of the maternal transcriptome. (A) Distribution of maximum fold changes (mFCs) in gene expression per gene between individual eggs (black, B left panel) and per gene within clutches (grey, B right panel). (B) Left panel, the maximum fold change (mFC) in gene expression between individual eggs, calculated as the difference per gene of the log intensity of the highest gene expression in any egg and the lowest log intensity of any other egg; Right panel, the maximum fold change within clutches was calculated per gene as the largest of the differences of the highest log intensity in any clutch (≈ mother) and the lowest log intensity in the same clutch (≈ mother). The red dashed line represents an absolute gene-expression mFC of 2. (C) Four illustrative examples of similar gene-expression in clutches from different mothers (color code see Fig 1A). Labels on the X-axes represent ‘Mother (clutch)’.

To get a first impression of the variability of the maternal transcriptome between individual eggs, we examined per gene the maximum fold change (mFC), i.e. the highest measured intensity divided by the lowest. When we apply a threshold of mFC = 2 (log2 = 1), about 50% of the 11,118 expressed genes have a higher mFC, hence the other half of the genes have a bandwidth of just mFC = 2 (Fig 2A and 2B, visualized as log mFC). Also, only 5% of the genes have a mFC > 4. This means that the majority of expressed genes has limited gene-expression variability over all eggs in the experiment (Fig 2C). Even more so, we were interested to see if the observed variation originates from differences between clutches or individual eggs. For this, we calculated the mFC for each gene per clutch (Fig 2A and 2B). As a result, 88% of the expressed genes showed a clutch mFC ≤ 2 and almost all genes (99.5%) have a clutch mFC ≤ 4. This means that the differences of mFC observed in all expressed genes are primarily caused by differences between mothers, whereas there is limited variability between eggs from each mother. The combination of limited variability in maternal gene expression between mothers, and a substantial difference between mothers points to a strict regulation during the assembly of the maternal transcriptome in *Zebrafish* oogenesis.

### Mother-related gene expression

**Fig 3:**
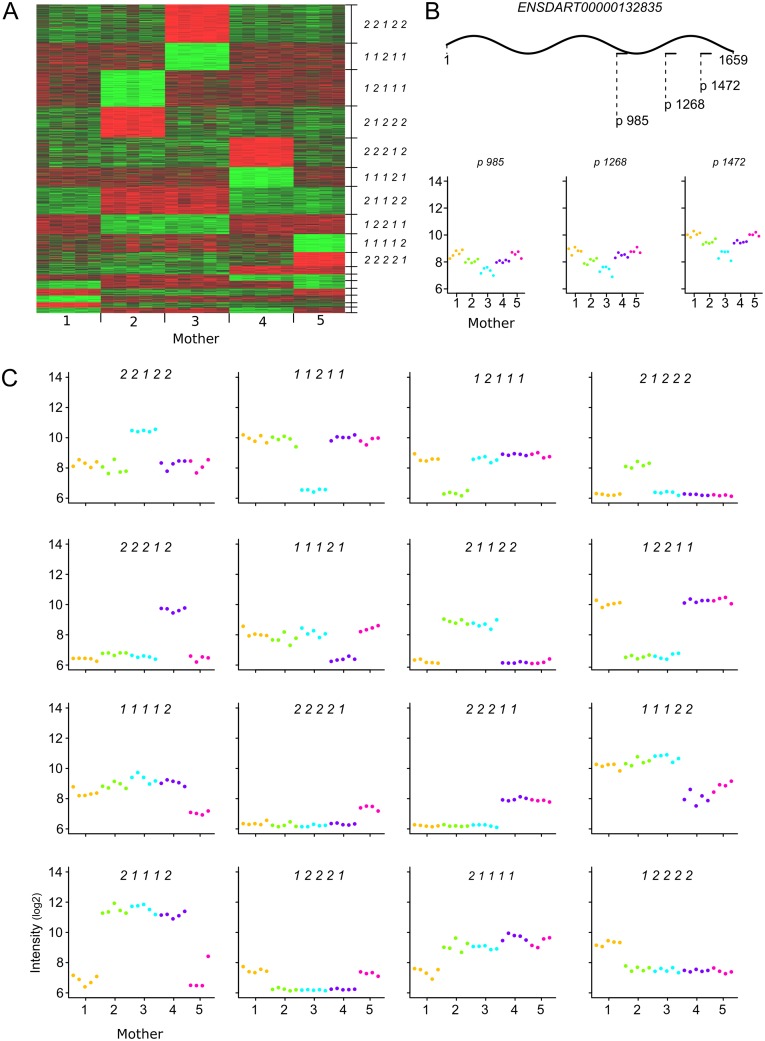
Obvious mother-specific maternal gene-expression. Maternal genes expressed at mother-specific distinct levels. (A) Heatmap showing 17 clusters of genes containing at least 25 genes that are expressed at a mother-specific or group-of-mothers specific level (see main text paragraph 3.4). The first 10 clusters are numbered by their relative gene-expression level between mothers as indicated by a 5-digit code (1 = high; 2 = low). In these 17 clusters only two levels were present because clusters with more than two levels contain less than 25 genes (S6 File) (B) Raw intensity data of three probes that query the transcript (ENSDART00000132835, pax1b-001) at positions: 985, 1268 and 1472 (color code: see Fig 1A). Labels on the X-axes represent ‘Mother (clutch)’, labels on the Y-axes represent log2 Intensity. (C) Illustrative examples of genes with mother-specific gene expression of the 16 clusters from A that have an inverse counterpart (color code see Fig 1A). Labels on the X-axes represent ‘Mother (clutch)’, labels on the Y-axes represent log2 Intensity.

To further understand the observed gene-expression differences between clutches, we evaluated the expression profiles of all expressed genes. Many genes showed an expression profile in which every egg from any mother showed a similar expression level, yet completely different than that of every egg from one or more of the other mothers. To rule out that this phenomenon was caused by point mutations in the probe target sequence in these genes, we checked several genes that were targeted by more than one probe. It showed that these profiles are, besides the expected variance in intensity due to variant probe-affinity, identical (Fig 3B). Thus the observed gene-expression differences represent genuine differences in mRNA presence.

Effectively, eggs from one mother express each gene at one specific level, which can be substantially different than that in eggs of other mothers. As there are five mothers in this study, up to five of these specific gene-expression levels are possible for each gene. Because a different number of mothers can show the same expression level, this amounts to many potential different gene-expression level combinations. We identified 2,224 genes (20%) displaying absolute distinct mother-specific gene-expression levels (cf. Materials and Methods), i.e. there is no overlap between the expression range of a gene in eggs from one or two mothers as compared to the expression range of the remaining eggs. These genes could be grouped in 174 gene clusters with a unique expression profile (Fig 3A, and S9 File). 17 clusters contained more than 25 genes, whereas 63 clusters existed of only one gene. The observed profiles (Fig 3C) show a remarkable similar gene expression within mothers as well as obvious different gene expression between mothers. We were concerned that the differences between clutches could be caused by eggs that are per mother in a different stage of maturation, activation, meiosis, or possibly decay. However, as all these processes are based on strict cellular programs, the samples should cluster in an ordered fashion, much as is shown in many embryonic programs [18,22,57]. The fact that the gene expression and eggs cluster like a checkerboard (Fig 3A), effectively rules out the possibility that these gene expression differences are caused differences in cell phase, but are truly reflecting relative differences in maternal mRNA presence as accomplished during oogenesis. The strict regulation of maternal gene expression within each mother is quite impressive.

### Differentially-expressed maternal genes

**Fig 4:**
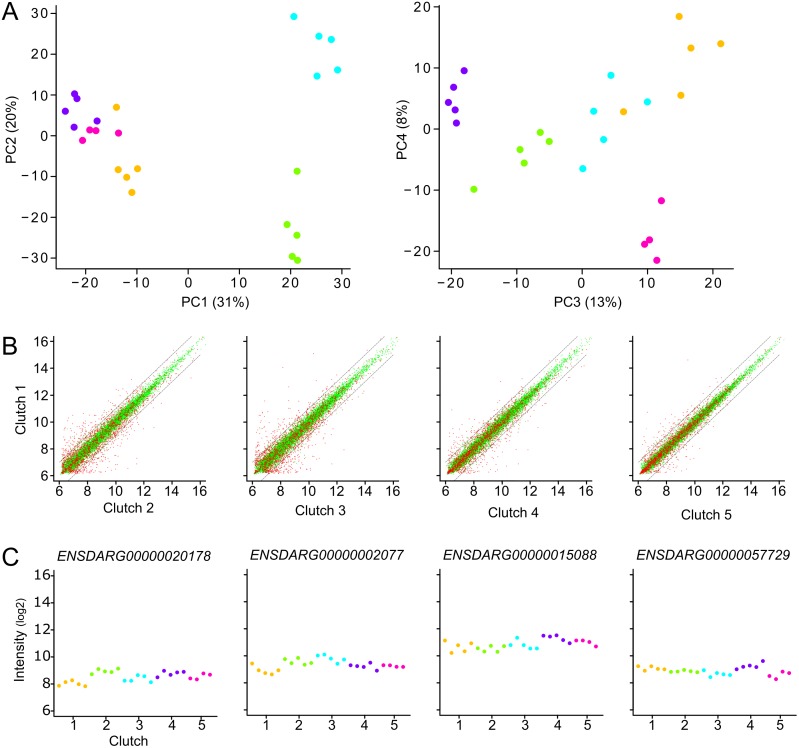
Subtle mother-specific maternal gene-expression. Overall low-variable maternal genes are almost all mother-specific differentially expressed. (A) Representation of the first four principal components of a PCA on the 11,118 expressed genes. (color code see Fig 1A). The contribution to the total variability is given as a percentage at the axes. (B) Scatterplots of the per gene average clutch expression value of the first clutch against the four other clutches. Red dots represent the 2,224 genes that show an absolute distinct mother-specific expression level; green dots represent all other expressed genes. Dashed lines represent a fold change of 2 between the two clutches (also S14 File). Labels on the Y-axes represent ‘Clutch 1’. (C) Illustrative examples of four mother-specific differentially-expressed genes that do not have an absolute distinct mother-specific expression. Labels on the X-axes represent ‘Mother (clutch)’, labels on the Y-axes represent log2 Intensity.

The above gene clusters were defined naïvely, as well as by absolute difference between mothers. In order to determine whether more genes bear a maternal gene-expression signature, we determined the differences in gene expression within and between mothers with a standard ANOVA (P< 0.05 at a false discovery rate of 5%) using ‘clutch’ as model factor. Remarkably, the ANOVA identified over 99% (11,005) of the genes to be differentially expressed (DEG) between any of the mothers (S10 File). Given the quite low mFCs per mother, this means that even in genes with very low variance in gene expression over all eggs, stricter regulation within each mother can be observed (Fig 4B and 4C). All the small mother-specific differences add up to a maternal-specific transcriptome that notably differs between clutches, which is illustrated by the PCA plots in which eggs from each mother convincingly cluster together and separate from those of other mothers (Fig 4A).

### Elucidating the mother-specific gene expression

Given the obvious maternal gene-expression patterns between mothers, a specific regulation of the maternal mRNA pool in the developing oocyte must be in place. As little is known about the underlying regulation mechanisms, we tried to elucidate the mother-specific maternal mRNA expression by analyzing the previously-defined 17 gene clusters with absolute gene-expression differences between mothers, for potential clues to their origin and function. As first perspective, we analyzed whether we would be able to associate the detected cellular processes that are overrepresented in the set of expressed genes (Fig 1D) to a mother-specific expression profile. If these cellular processes were to be associated with gene sub-clusters, then this would immediately show when mapped onto a heatmap of all expressed genes. However, mapping several of these clusters showed no tendency for these gene sets to associate with gene sub-clusters; the only relation we could find was that many of these expressed gene-sets have a high expression level, which is to be expected taken into account the stock characteristic of maternal RNA at this developmental stage (data not shown).

Additionally, the potential function of the 17 mother-specific gene clusters was investigated, using all expressed genes as a background, with over-representation using KEGG and GO gene sets. Only relatively weakly associated gene-sets were found for these gene clusters (S11 File).

We also mapped the Aanes *et al*. defined clusters [16] to our mother-specific gene clusters, but did not find any obvious relation. Which is to be expected as the Aanes clusters are defined by degradation and activation profile of RNA in early embryogenesis, rather than by the RNA production during oogenesis. Similarly, only a few of the mother-specific gene clusters showed overrepresentation of the mentioned 3’ UTR located hexamers and cPEs (S12 File). Also the most overrepresented set in the expressed vs. non-expressed comparison, i.e. the set of embryonic CPEs, was not overrepresented in any of the mother-specific gene clusters. So in this respect there seems to be no correlation between the assembly of the mother-specific genes RNA pool and stabilization and/or activation via polyadenylation of these mRNA molecules.

**Fig 5:**
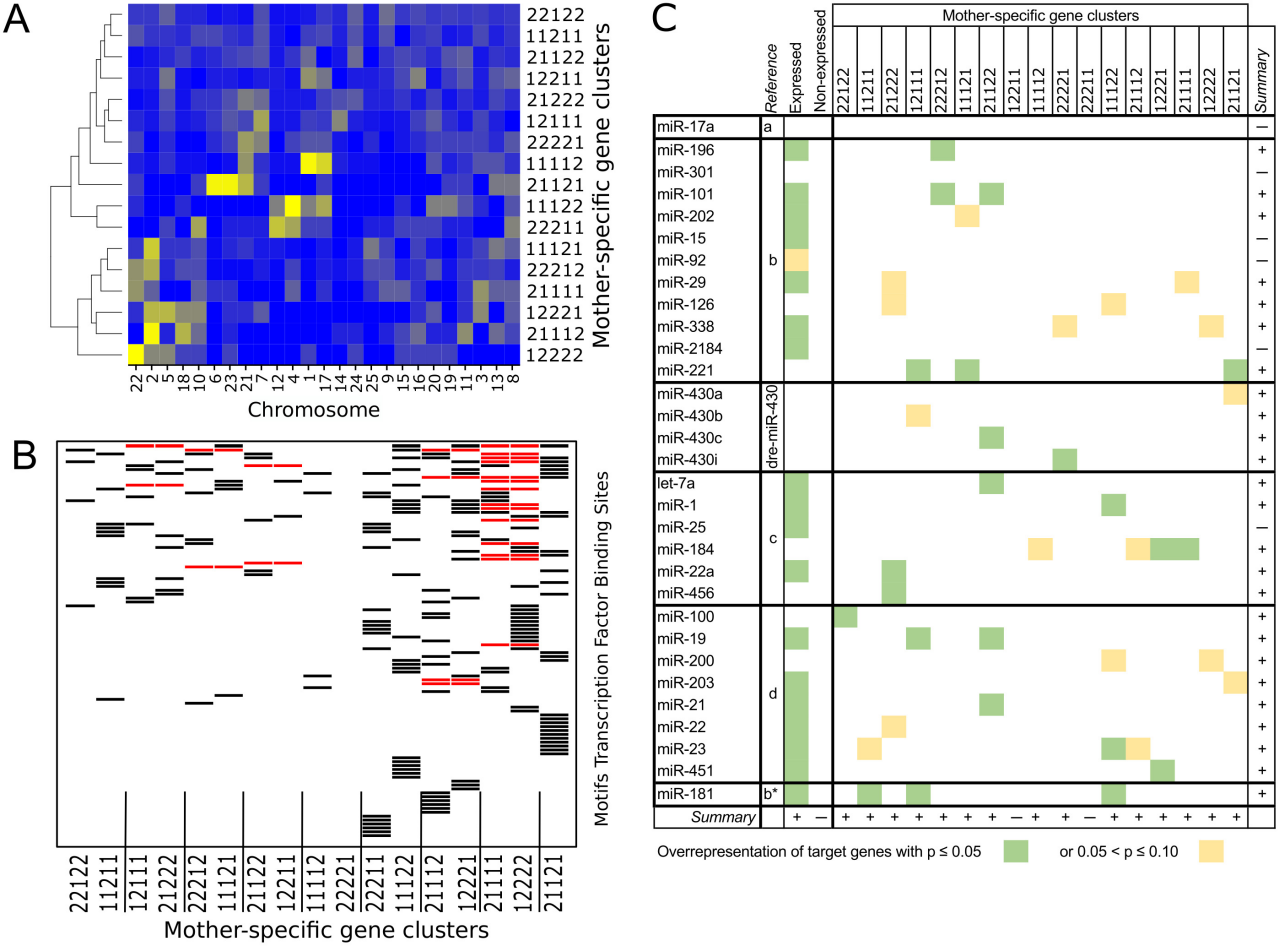
Characteristics of mother-specific maternal gene expression. Characteristics of the 17 mother-specific gene clusters defined by relative gene-expression differences (see Fig 3A). (A) Clusterogram of chromosome location of the genes per cluster. Indicated is the relative occurrence of genes on a chromosome: dark blue, low; bright yellow, high. (B) Schematic representation of the overrepresented, TRANSFAC-defined transcription-factor binding sites (TFBS) in the upstream regions of the genes per cluster. Each horizontal line denotes a specific TFBS (specified in the same order from top to bottom in S13 File). A bar indicates that a specific TFBS is found overrepresented in the specified mother-specific gene cluster. Red bars indicate that a specific TFBS is found overrepresented in both related, inversed gene clusters. (C) Representation of the overrepresented miRNA target sequences in the transcript sequences of the genes per cluster, as well as for the expressed and non-expressed genes. The targets for miRNAs reported in the following references were tested: a *Abramov et al*. [51], b: *Juanchich et al*. [52], c: *Yao et al*. [53], d: *Wei et al*. [54], b* whereas in [52] the minor form of miR-181 was reported, here the targets for the major form of miR-181 were tested, because in TargetScanFish 6.2 no targets for miR-181* are available.

Another perspective, the position of the cluster genes in the genome (S9 File) was examined, as it is known that the organization of chromosomes is often important in gene expression[58]. There is a clear non-random distribution of the cluster genes over the chromosomes (S3 Fig). As can be read from the heatmap of the percentage of genes in a particular cluster that maps to a specific chromosome (Fig 5A), each mother-specific gene cluster has a different chromosome distribution of its genes. Yet, after clustering the chromosome distribution of the gene clusters, it turned out that the gene clusters that have an inverse expression profile over the mothers cluster together. This means that for instance the gene cluster in which the genes of mother 1 were expressed *higher* than in all other mothers show a chromosome distribution similar to the inverse gene cluster in which the genes of mother 1 were expressed *lower* than in all other mothers (Fig 5A). Out of the eight possible inverse cluster combinations, six clustered as a pair in our heatmap (Fig 5A). So, clusters of genes with a specific expression profiles over mothers, both up and down, show a similar correlation with chromosome location, which strongly suggests a genome-based regulation mechanism.

Also, the presence of overrepresented transcription-factor binding sites (TFBS) in the upstream region of the cluster genes was studied. Many potentially relevant TFBSs (101) were found to be specific for genes in the identified mother-specific gene clusters using all the genes in the mother-specific gene clusters as a background (Fig 5B, S13 File). Moreover, as with chromosome location, several (21) of these TFBS were found, sometimes exclusively, in associated inversed clusters (Fig 5B). Altogether, these findings point towards a role of transcription factors in the observed regulation of the maternal gene-expression.

Finally, as several miRNAs were strongly associated with maternally-expressed genes, we investigated the relation between the mother-specific gene clusters and several miRNAs involved in oogenesis and early-embryogenesis. For this we looked whether the target genes of a specific miRNA were overrepresented in a gene cluster. The results indicate a strong relation between specific miRNAs and specific gene clusters (Fig 5C and S7 File). Of the 17 gene clusters, 15 could be associated with a tested miRNA and reciprocally; of the 31 tested miRNA, 25 showed an association with any of the gene clusters (Fig 5C). Moreover, it seems that the interaction was rather specific in that most miRNAs just correlated with one or two gene clusters. Here, the reciprocal gen-expression clusters do not display co-regulation. Given the well-known role of miRNA in gene-expression regulation, it seems that miRNAs have an important role in the emergence of the observed mother-specific gene clusters.

## Conclusions

The extent of the conclusions in our study is defined by the fact that in this experimental set-up, mother and clutch are confounding factors. We do not know how subsequent clutches from a mother relate to each other with respect to their maternal transcriptome. At the same time, in this study only relative gene-expression differences of genes represented by a probe on the microarray are analyzed. Substantial variance in RNA yield was observed after isolation, but by lack of proper means to control this process, it is impossible to determine whether these differences are actually present in the eggs, are a result from the isolation procedure, or are merely caused by a different rRNA content of the eggs. Nevertheless, the relative gene-expression differences found in this study are very convincing, because they are observed between clutches, while at the same time the gene-expression within a clutch is highly similar. Additionally, given the total lack of order in the detected mother-specific gene-clusters it is not likely that the observed gene-expression differences originate from different stages of maturation, meiosis, or activation of the studied eggs. One outcome of our study, the consistency of the maternal transcriptome within each clutch, means that pooling of mature oocytes or eggs for gene-expression studies will likely not severely affect the outcome, although replicate pools will be needed to statistically determine the mother-specific differences.

There are two main conclusions that emerge from this study. First and foremost, it seems that the maternal transcriptome in *zebrafish* eggs is incredibly tightly organized; half of all expressed genes shows maximally a lower than twofold difference between any of the 24 profiled transcriptomes. Even more, the maximum gene-expression difference within clutches is for 88% of the expressed genes lower than twofold. It seems improbable that such a tight regulation of gene-expression levels is regulated by transcription alone, so it is likely that a fine-tuned system of transcription, stabilization and degradation is in place during oogenesis. Although one could argue that embryogenesis is a tightly programmed process and as such it is logical that maternal RNAs are also tightly regulated, the extent of similarity between the transcriptomes over mothers has surprised us.

The second main conclusion is that the subtle differences we find are highly specific for mother, given that over 99% of the expressed genes are differential with respect to mother. This aspect is also illustrated by the fact that all clutches are convincingly separated in a principal component analysis of the expressed maternal transcriptomes. This is even more remarkable because the mothers studied here are siblings, in which we may assume limited genetic variation. Nevertheless, we cannot exclude that genomic variation might be a partial cause of this mother-specific behavior, but from the example in Fig 3B we learn that it cannot be the only cause of this. Although we can only speculate why these clusters exist, their specific occurrence with respect to chromosome localization indicates a functional cause, a hypothesis further strengthened by the occurrence of overrepresented clusters with inversed gene expression on identical chromosomes. Also the co-occurrence of the same transcription factor binding sites in promoters of genes in the mother-specific clusters and their inverse counterpart hints on a biological function for the subtle differences in the transcriptomes of different clutches. The analysis of miRNAs provides the most convincing evidence of gene-expression regulation, in that we were able to establish a relation between the mother-specific gene clusters and specific miRNAs. Although these functional analyses were limited to identifiable gene-clusters, one has to realize that these clusters extend beyond the absolute between-mother differences in which we defined them. Virtually all genes were found to display mother-specific gene-expression differences, so all genes are under the control of many factors that purposely result in a mother-specific signature in maternal *zebrafish* transcriptomes.

Altogether, the maternal transcriptome composition of mature *zebrafish* oocytes seems to be tightly regulated in a mother-specific manner, controlled during oogenesis by many factors, such as chromosome conformation, transcription factors and miRNAs.

## Supporting Information

S1 Fig Data-quality control after normalization. (A) All raw intensity data were centered on the array average. Shown is the per probe correlation of the used scaling factors and the centered intensities vs. the standard deviation of these centered intensities. 194 suspect probes with a high anti-correlation and a high standard deviation are indicated with red dots. (B) Distributions of all gene-expression data (black) and the gene expression of the 194 red probes from A (blue) in a similar, but separate 36 single eggs microarray experiment in which a different protocol was applied. In this experiment, shortly described below, overall very low signal intensities were observed. We attribute these low intensities to the polyadenylation step on total fragmented RNA; i.e. due to the very large amount of molecules to be polyadenylated the polymerase was exhausted prior to generating sufficiently long poly(A) tails to all mRNAs. However, mRNA molecules with native long poly(A) tails would still be able to produce a sufficient signal. In this experiment the 194 suspect genes from the experiment in the current 24 egg study show a substantially higher expression value than most other genes and hence will be in this category of transcripts with a native long poly(A) tail. We reasoned that the performance and the efficiency of the polymerase is likely a major reason for the sample-to-sample variability and is a major factor on which the data in the 24-egg is normalized. However, if mRNAs are already polyadenylated such a normalization likely produces spurious intensity data for these genes. Therefore we removed these genes from further analysis. *Description of the 36-egg array experiment of Fig S1B*: The experimental setup of the experiment shown in Fig S1 is similar to the 24 egg experiment discussed in this article. Below is a short specification of this experiment: • Array used: NimbleGen custom microarray with a design to 24 egg experiment [39]; • Biological material: 36 eggs, similar to 24 egg experiment (cf. Materials and Methods); • Protocol used: after isolation of total RNA similar to 24 egg experiment: fragmentation followed by polyadenylation and IVT similar to 24 egg experiment; • Microarray procedure: according to manufacturer’s protocol (Nimblegen); • Scanning and data extraction: similar to 24 egg experiment.
(PDF)

S2 Fig Maternal gene expression in *zebrafish* eggs. (A) Distribution of the gene-expression intensity variance of raw expression data. The variance cut-off is indicated by a dotted vertical line at -3.74 (cf. Material and Methods). (B) Distribution of the gene-expression intensity of the variance categories. (C) Distribution of transcripts by the number of eggs in which each was called present based on the gene-expression variance cut-off from A. Grey: Transcripts that are called present in at least 4 eggs as a definition for the category: “Expressed” in this study. (D) Distribution of the gene-expression intensity of the expressed categories.
(PDF)

S3 Fig Chromosomal distribution of the genes in the 17 mother-specific gene clusters. Each page shows the schematic representation of the chromosome location of genes (red arrowhead) from a mother-specific gene-expression cluster as indicated by a 5-digit code (1 = high; 2 = low) in the upper right corner of the page.
(PDF)

S1 File RIN values and Yields of from the 24 *zebrafish* eggs. (XLSX)

S2 File Capture oligo sequences. (XLSX)

S3 File Ribo-depletion protocol. (DOCX)

S4 File Genes with a high anti-correlation with a calculated scaling factor were removed from further analysis. (XLSX)

S5 File List of expressed and non-expressed genes (XLSX)

S6 File Overlap of this study with the study of Aanes *et al*. (XLSX)

S7 File Over-representation in expressed, non-expressed and mother-specific cluster genes for targets of miRNAs reported of importance in oogenesis and early embryogenesis. (XLSX)

S8 File Over-representation of KEGG pathways and Gene Ontology categories in expressed and non-expressed genes. (XLSX)

S9 File Genes showing absolute distinct mother-specific gene-expression levels. (XLSX)

S10 File Differentially expressed genes with p< 0.05 at a false discovery rate of 5% using ‘clutch’ as model factor. (XLSX)

S11 File Over-representation of KEGG pathways and Gene Ontology categories in mother-specific gene-expression clusters. (XLSX)

S12 File Enrichment of hexamer motifs, PASs and PBEs in 3' UTRs of mother-specific gene-expression clusters. (XLSX)

S13 File Transcription factor binding motifs found in the promoters of the mother-specific gene-expression clusters. (XLSX)

S14 File Scatterplots of the per gene average clutch expression values. Red dots represent the 2,224 genes that show an absolute distinct mother-specific expression level; green dots represent all other expressed genes. Dashed lines represent a fold change of 2 between the 2 clutches.
(PDF)
