# Supplementary figures and images for: Mother-Specific Signature in the Maternal Transcriptome Composition of Mature, Unfertilized Zebrafish Eggs
Source: PLoS One. 2016 Jan 22;11(1):e0147151. doi: 10.1371/journal.pone.0147151 (PMC4723340; doi:10.1371/journal.pone.0147151)

**A**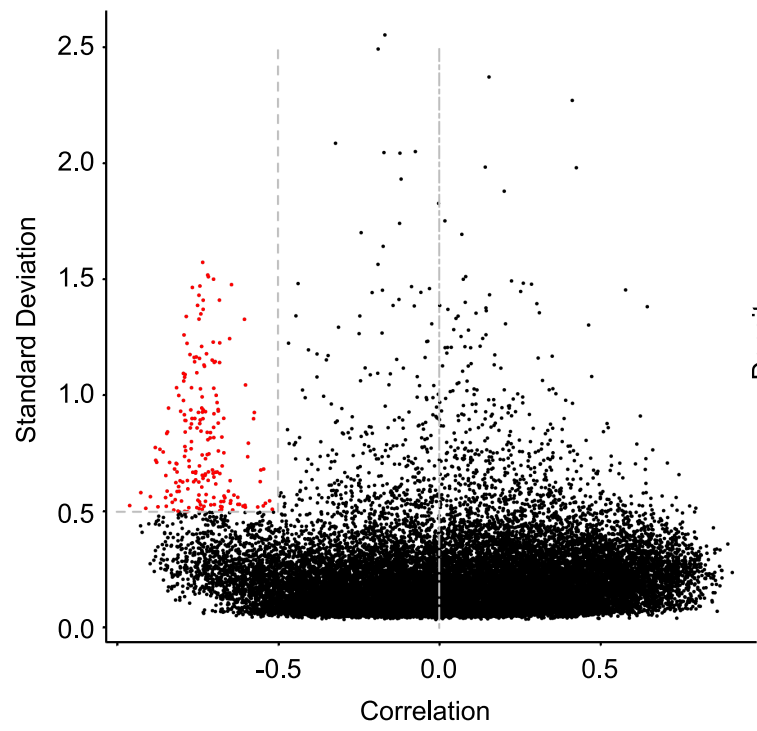**B**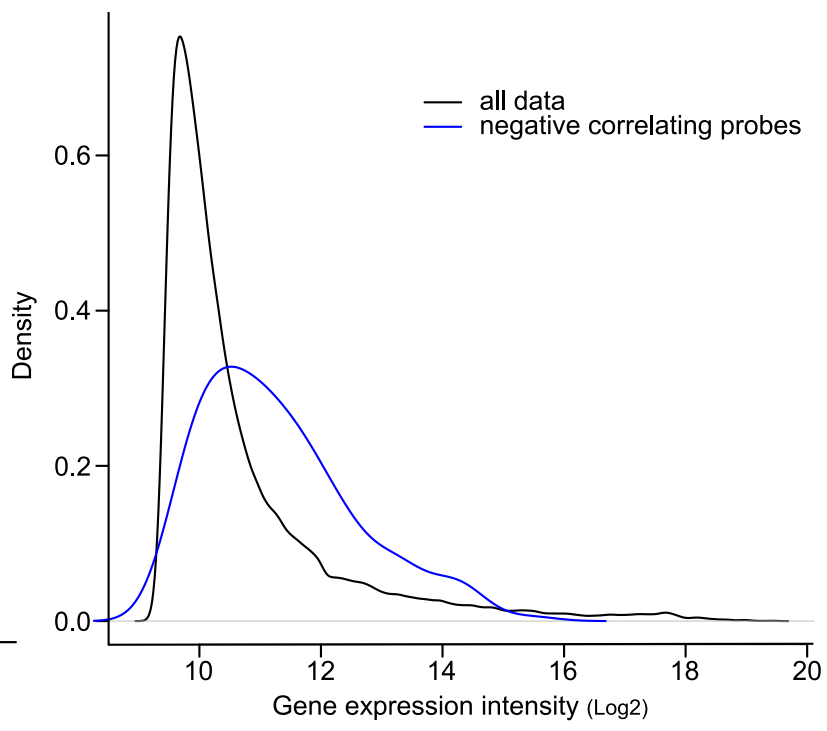

Supplement: S1 Fig — (A) All raw intensity data were centered on the array average. Shown is the per probe correlation of the used scaling factors and the centered intensities vs. the standard deviation of these centered intensities. 194 suspect probes with a high anti-correlation and a high standard deviation are indicated with red dots. (B) Distributions of all gene-expression data (black) and the gene expression of the 194 red probes from A (blue) in a similar, but separate 36 single eggs microarray experiment in which a different protocol was applied. In this experiment, shortly described below, overall very low signal intensities were observed. We attribute these low intensities to the polyadenylation step on total fragmented RNA; i.e. due to the very large amount of molecules to be polyadenylated the polymerase was exhausted prior to generating sufficiently long poly(A) tails to all mRNAs. However, mRNA molecules with native long poly(A) tails would still be able to produce a sufficient signal. In this experiment the 194 suspect genes from the experiment in the current 24 egg study show a substantially higher expression value than most other genes and hence will be in this category of transcripts with a native long poly(A) tail. We reasoned that the performance and the efficiency of the polymerase is likely a major reason for the sample-to-sample variability and is a major factor on which the data in the 24-egg is normalized. However, if mRNAs are already polyadenylated such a normalization likely produces spurious intensity data for these genes. Therefore we removed these genes from further analysis. Description of the 36-egg array experiment of Fig S1B: The experimental setup of the experiment shown in Fig S1 is similar to the 24 egg experiment discussed in this article. Below is a short specification of this experiment: • Array used: NimbleGen custom microarray with a design to 24 egg experiment [39]; • Biological material: 36 eggs, similar to 24 egg experiment (cf. Materials a [file pone.0147151.s001.pdf]

**A**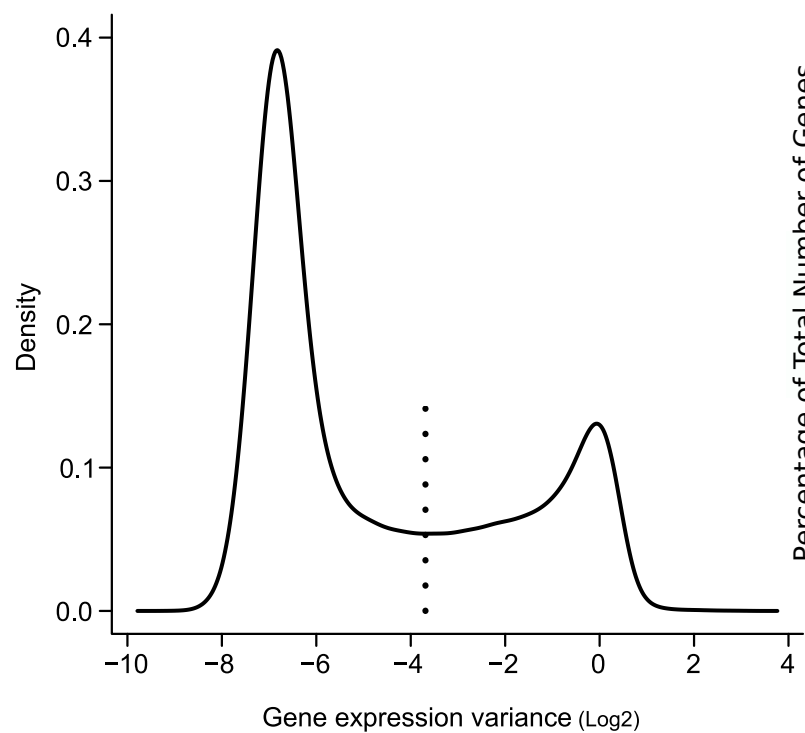**B**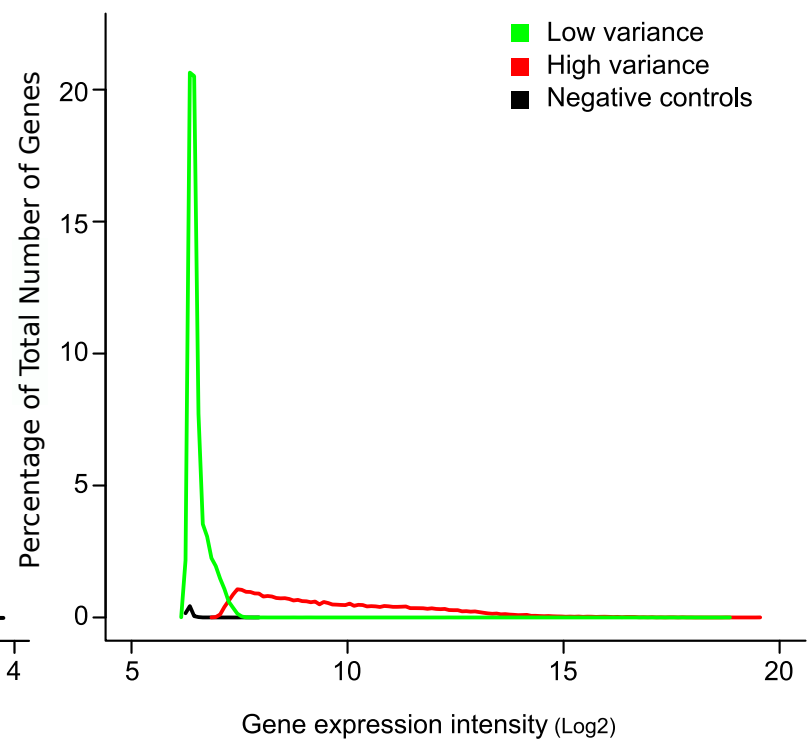**C**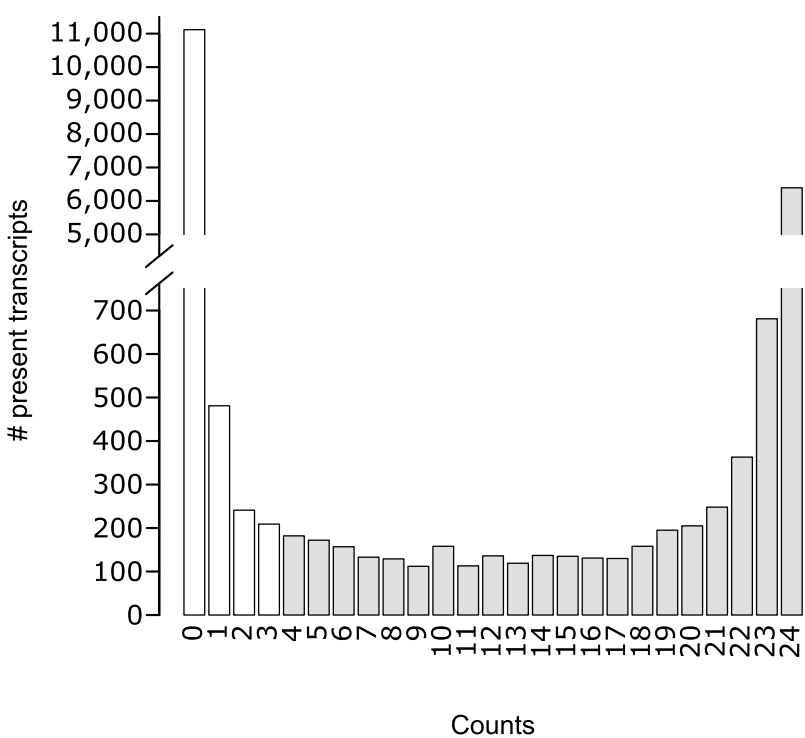**D**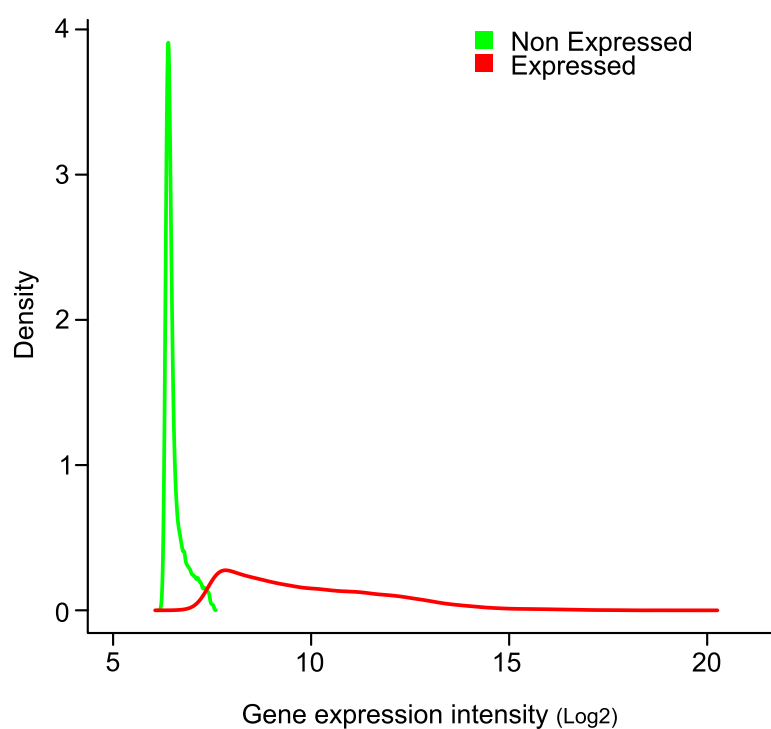

Supplement: S2 Fig — (A) Distribution of the gene-expression intensity variance of raw expression data. The variance cut-off is indicated by a dotted vertical line at -3.74 (cf. Material and Methods). (B) Distribution of the gene-expression intensity of the variance categories. (C) Distribution of transcripts by the number of eggs in which each was called present based on the gene-expression variance cut-off from A. Grey: Transcripts that are called present in at least 4 eggs as a definition for the category: “Expressed” in this study. (D) Distribution of the gene-expression intensity of the expressed categories. (PDF) [file pone.0147151.s002.pdf]

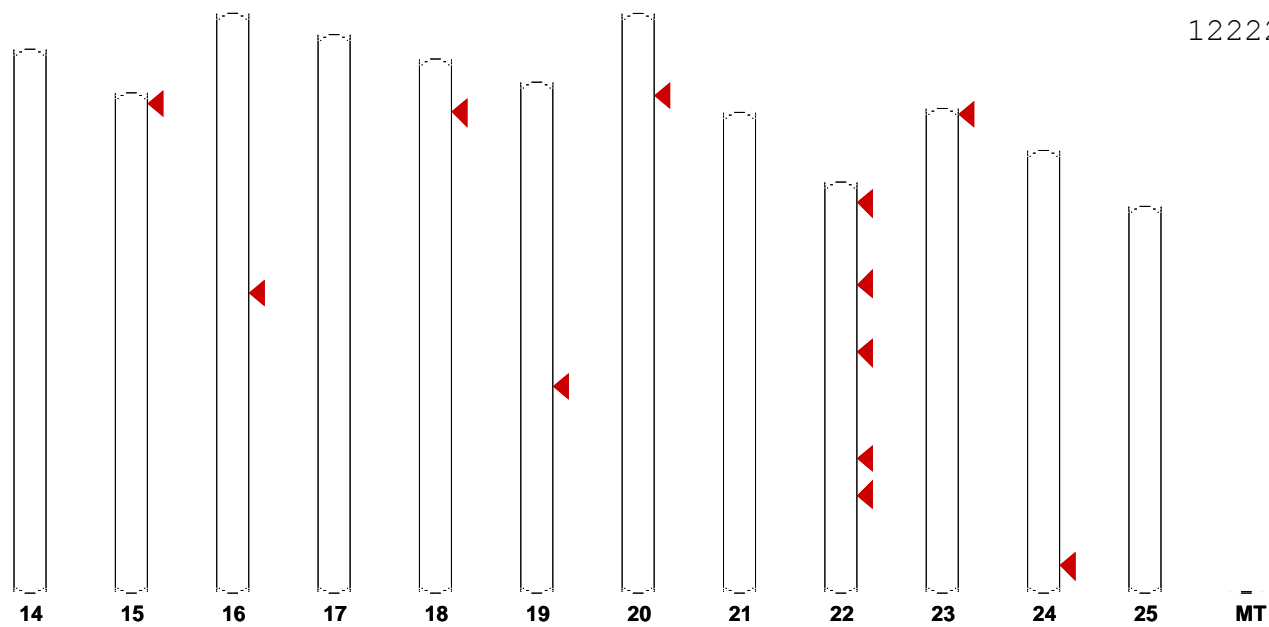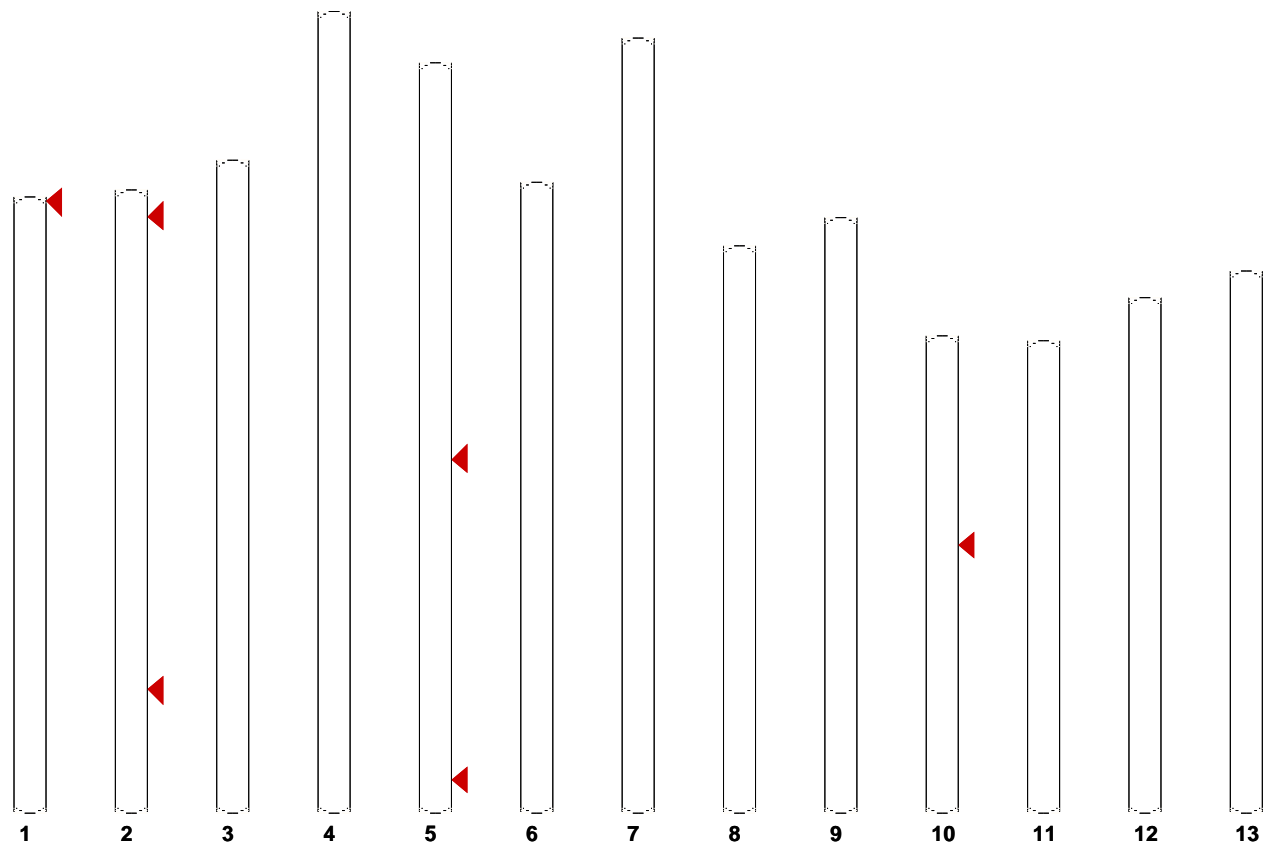

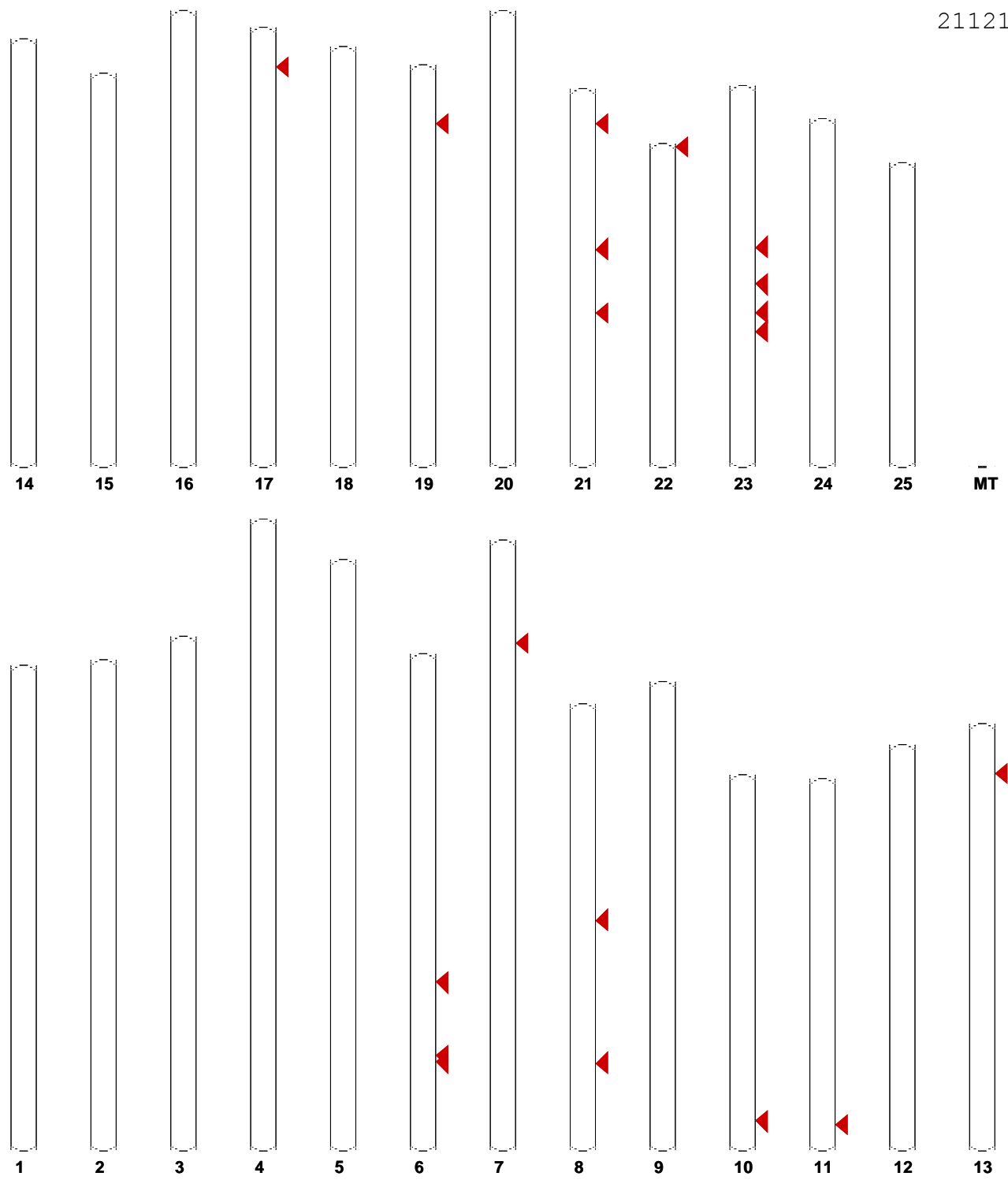

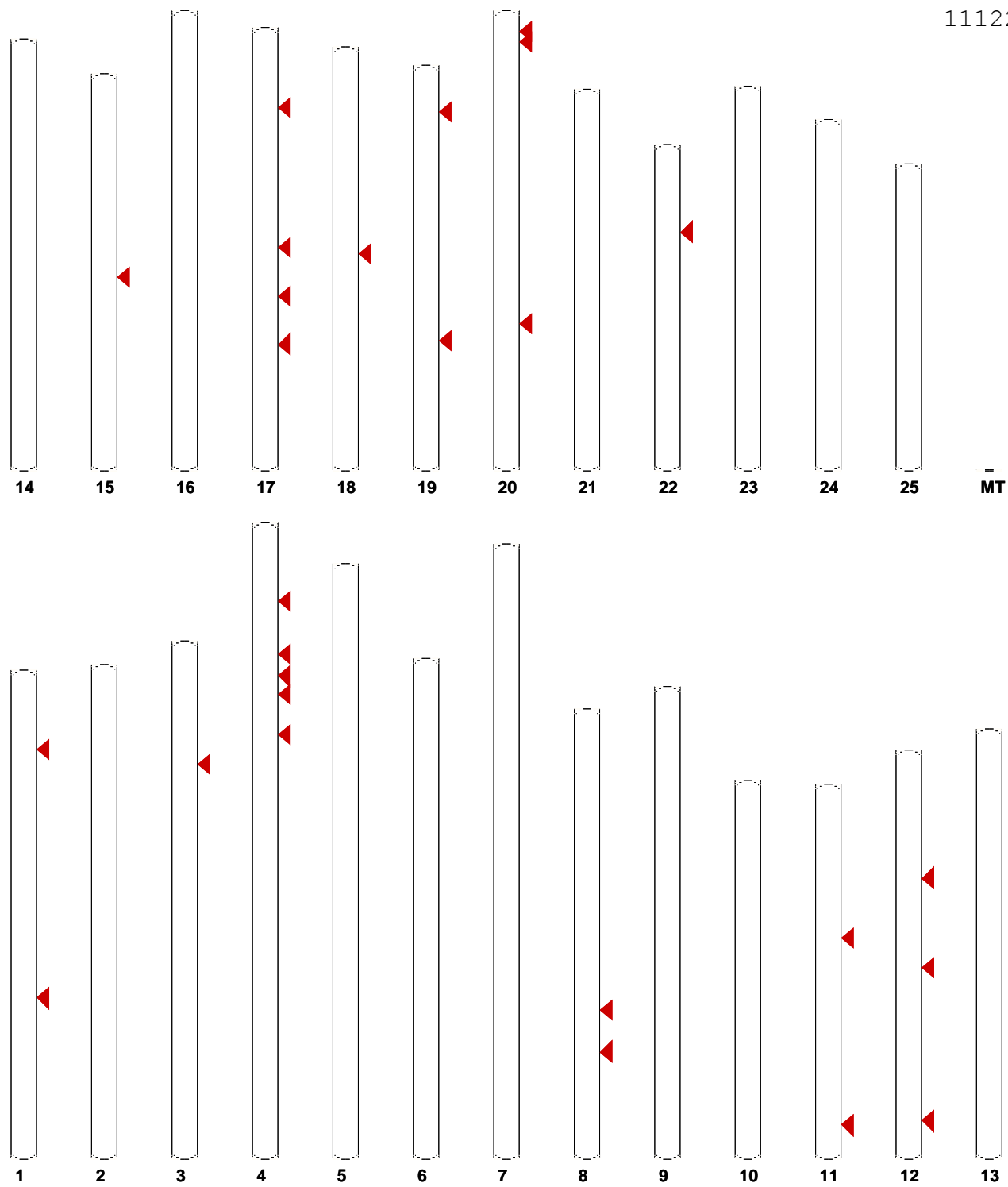

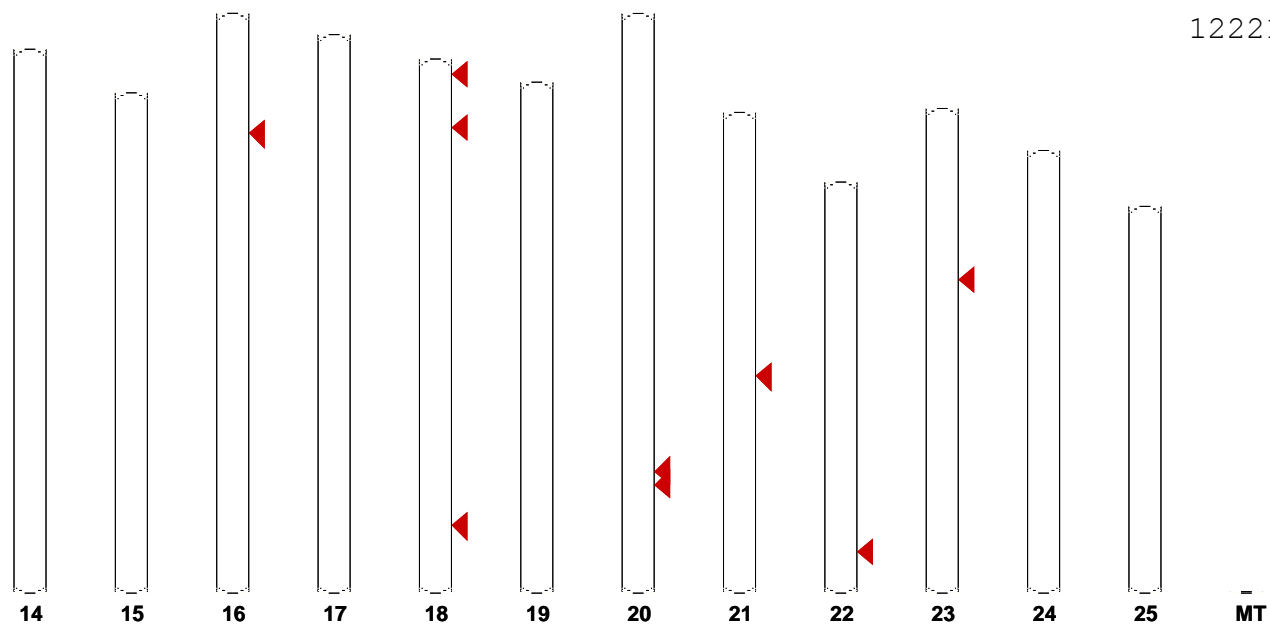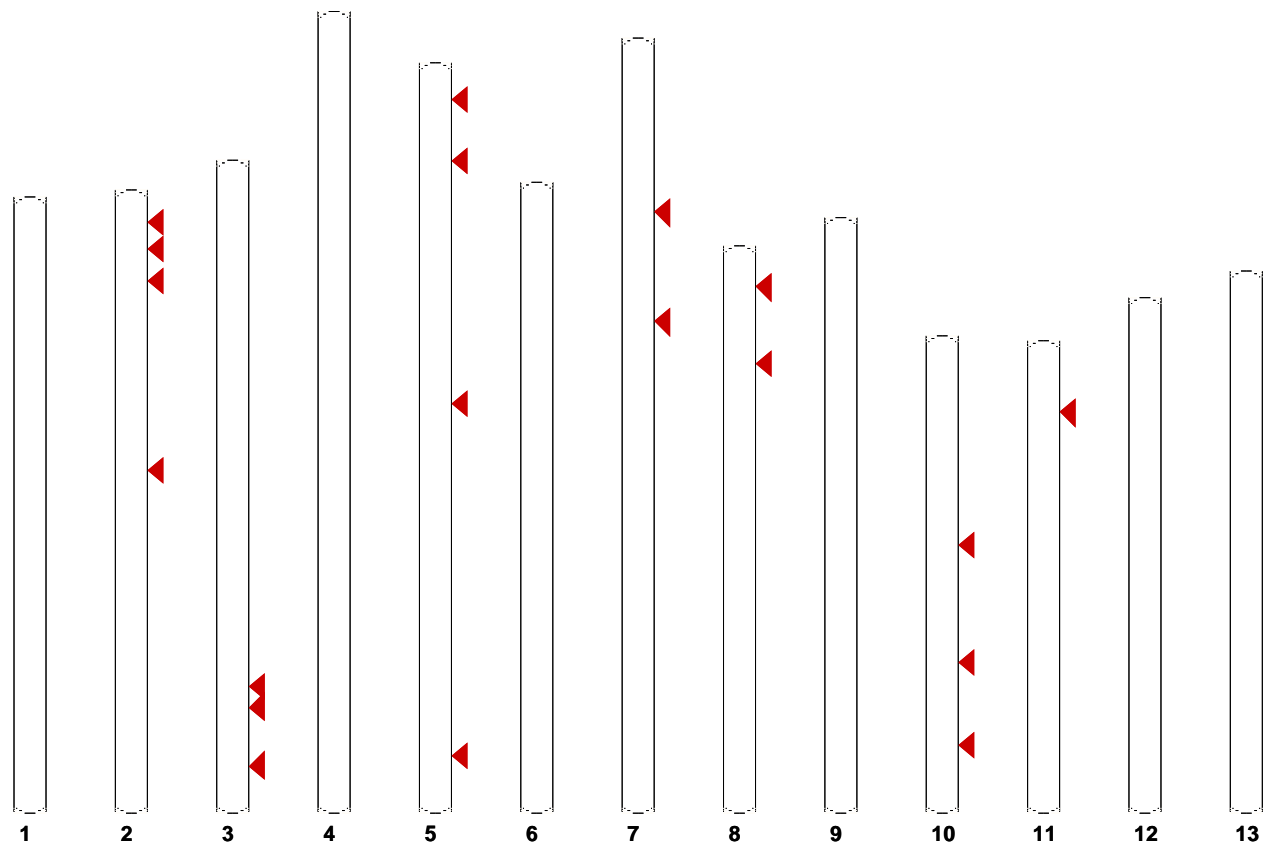

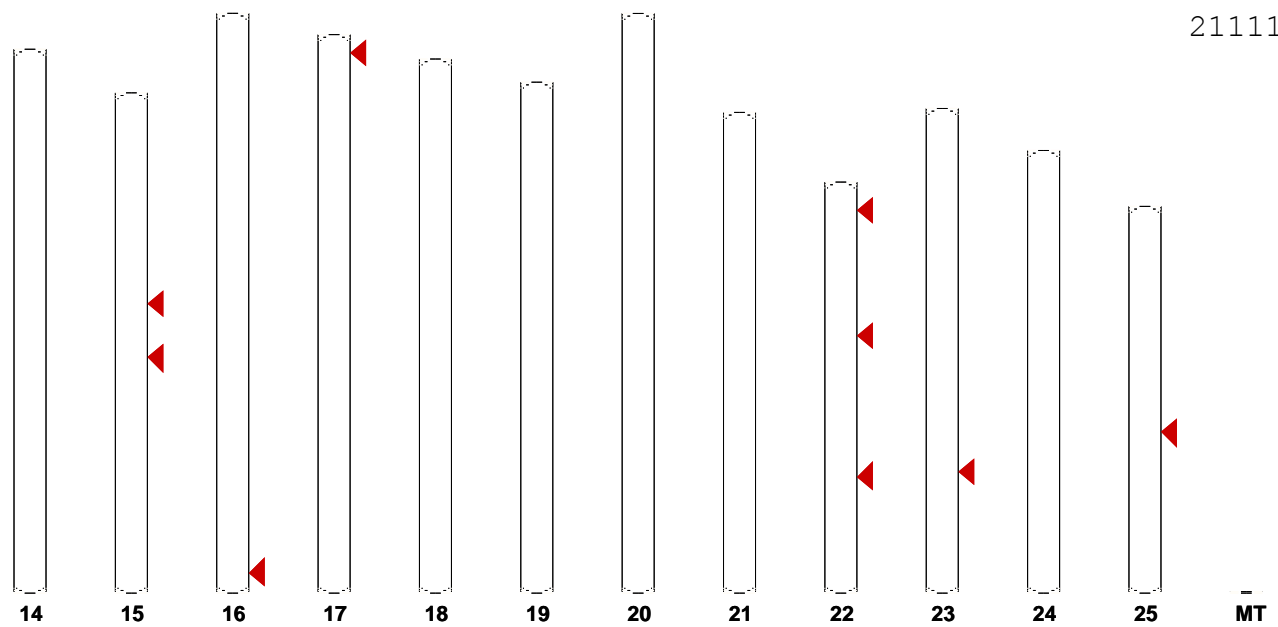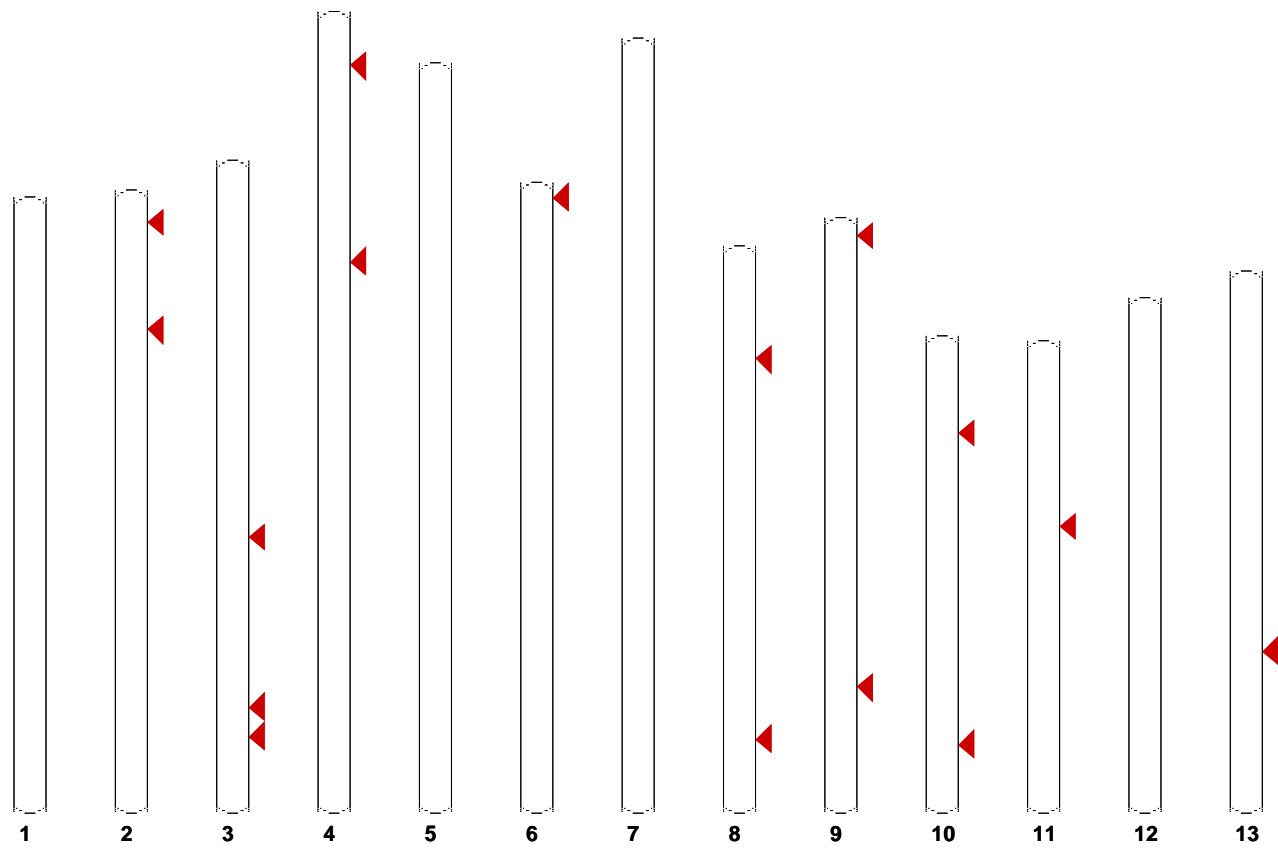

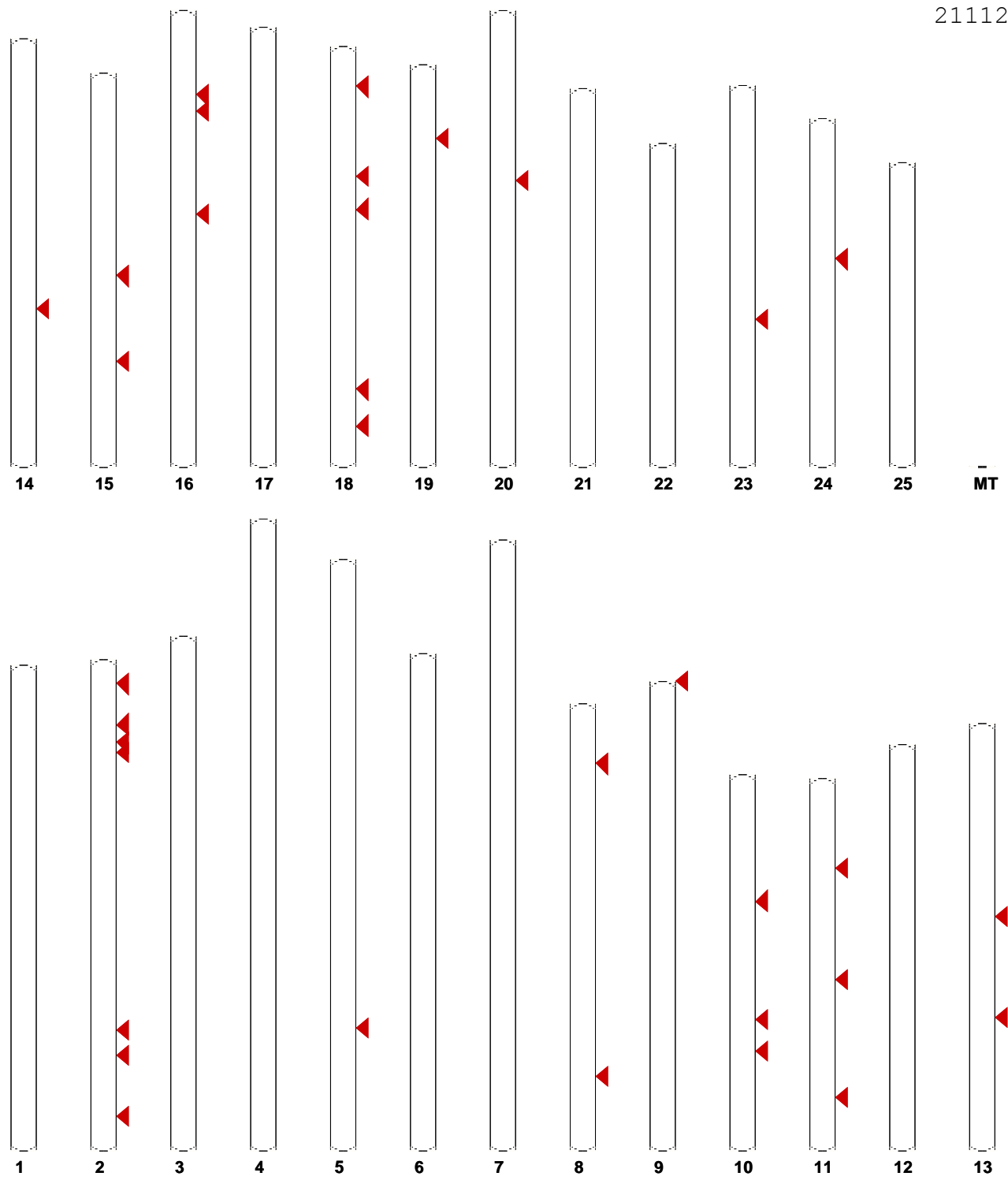

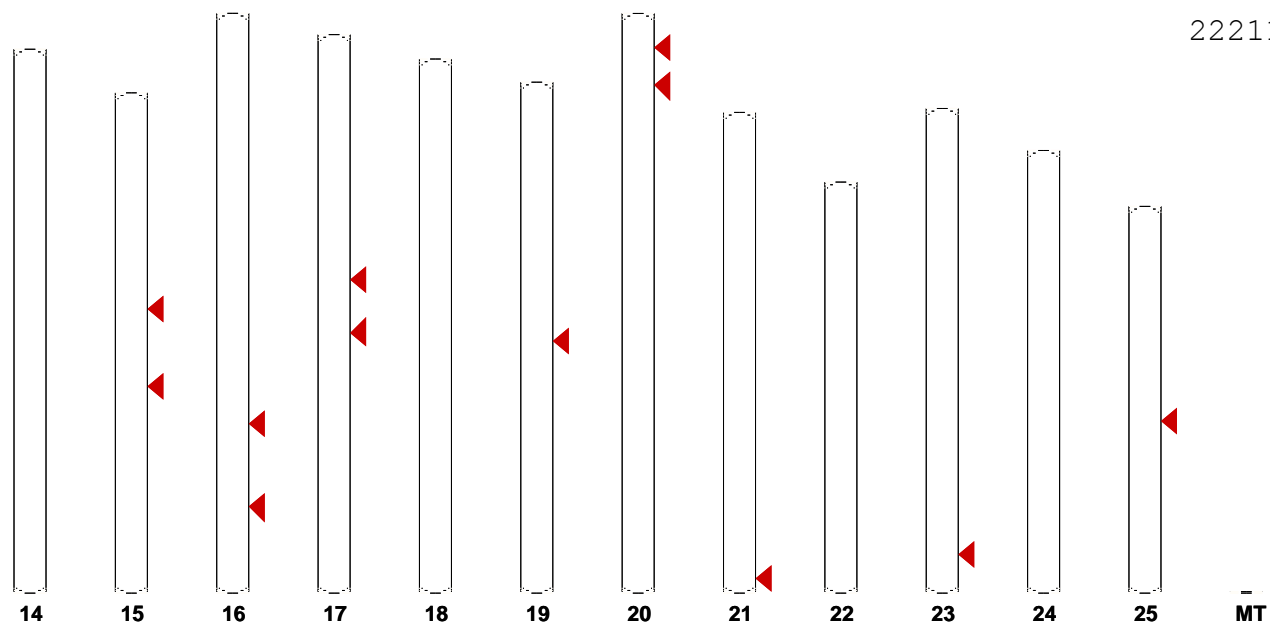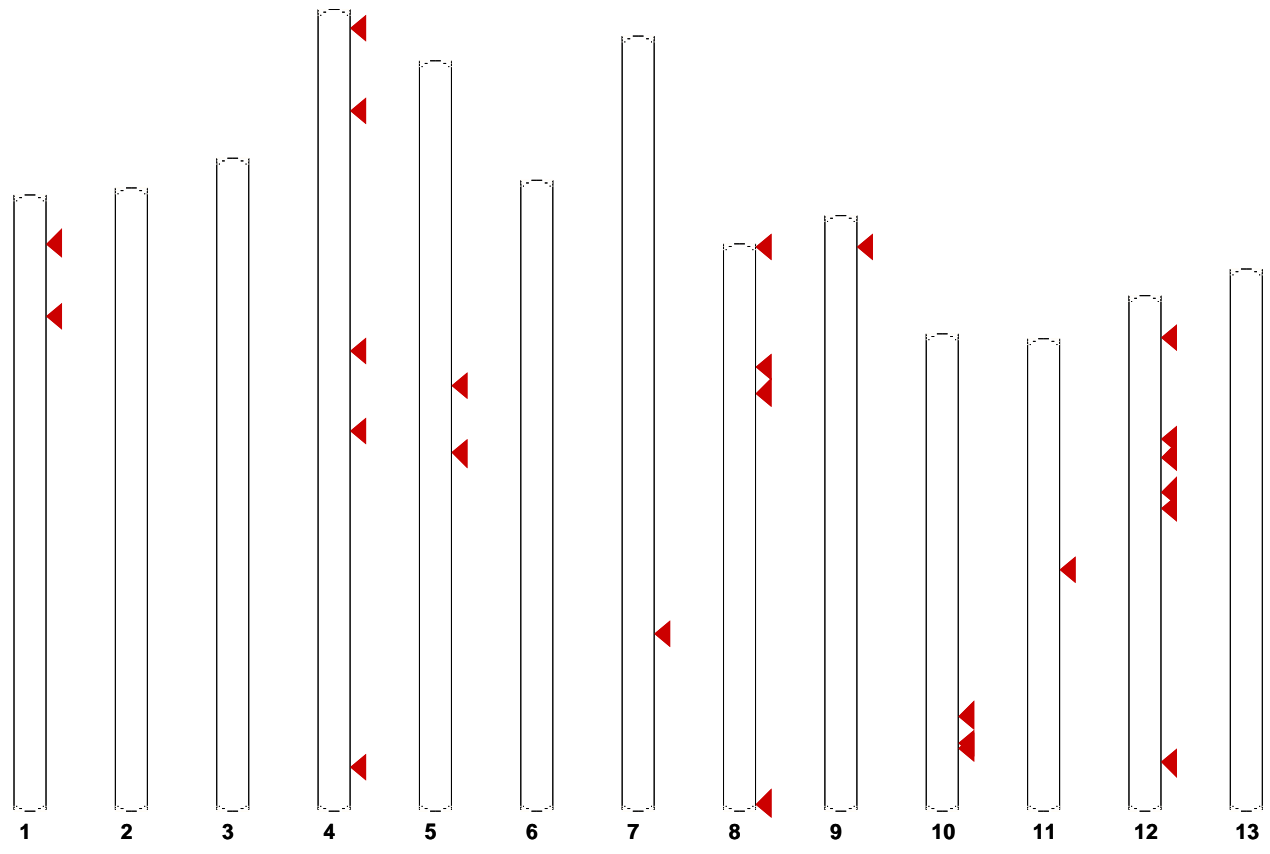

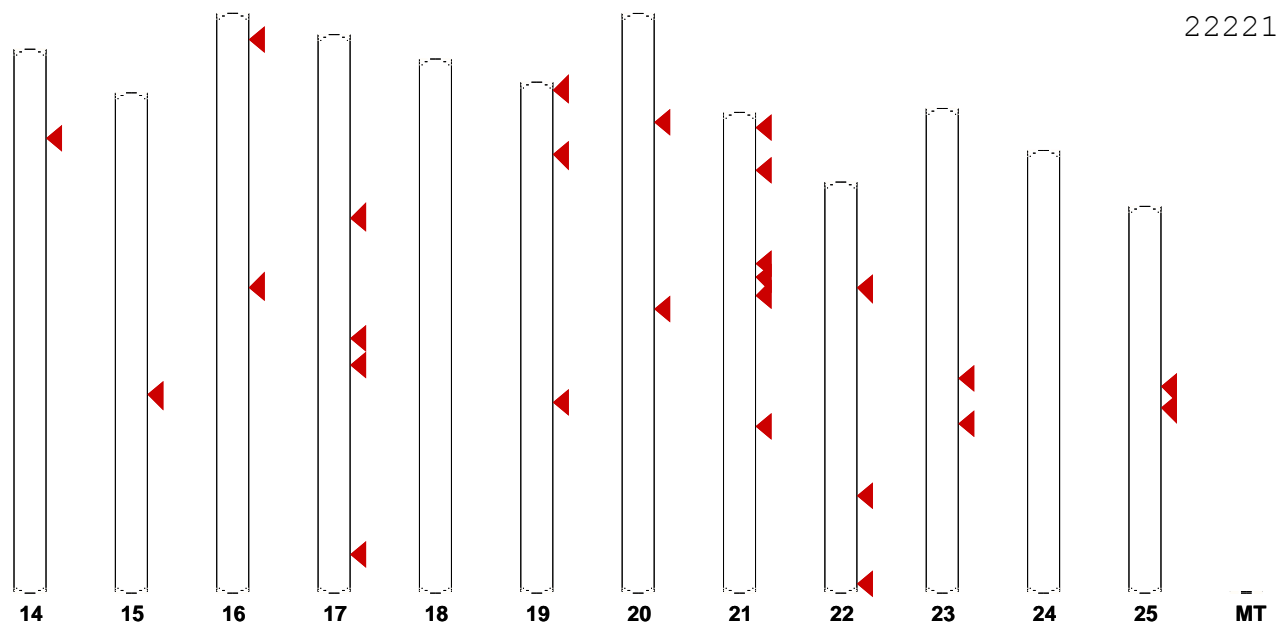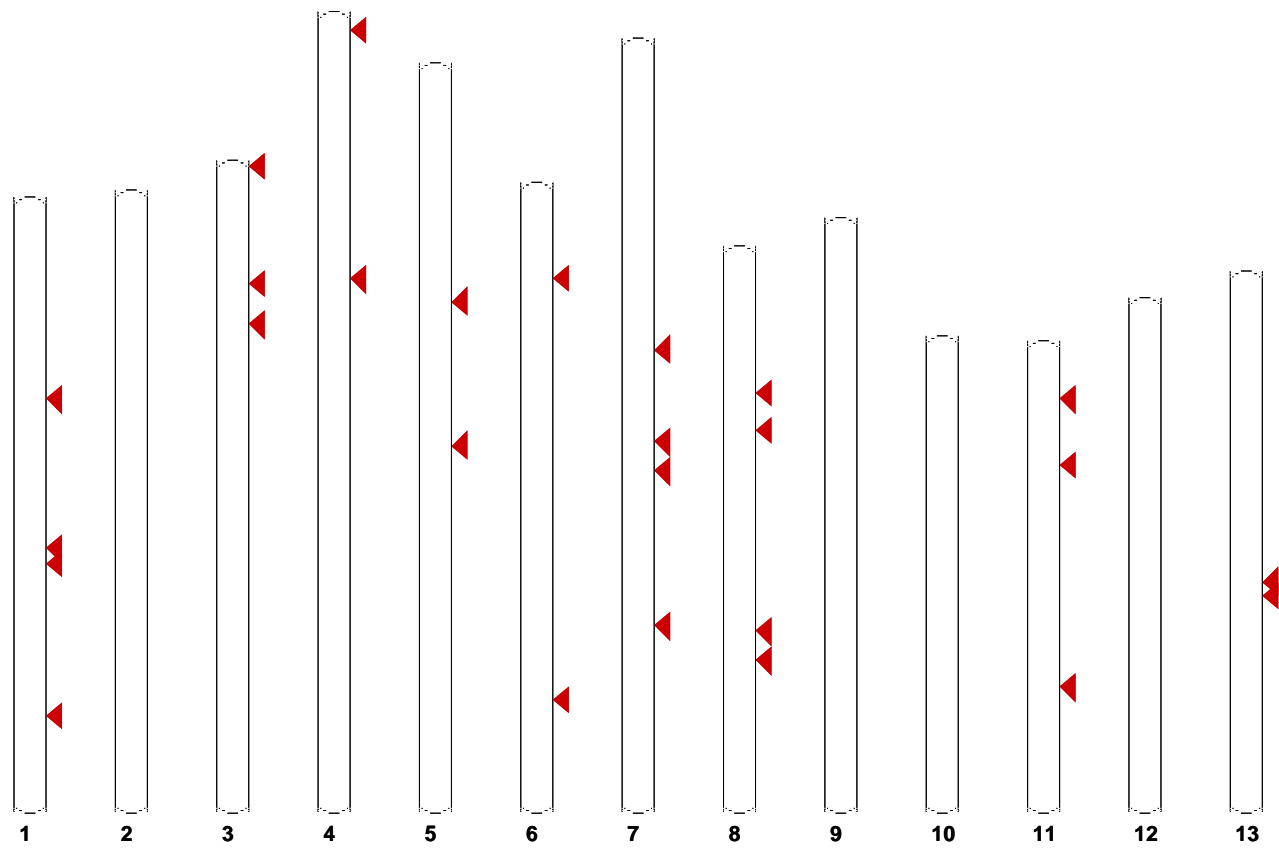

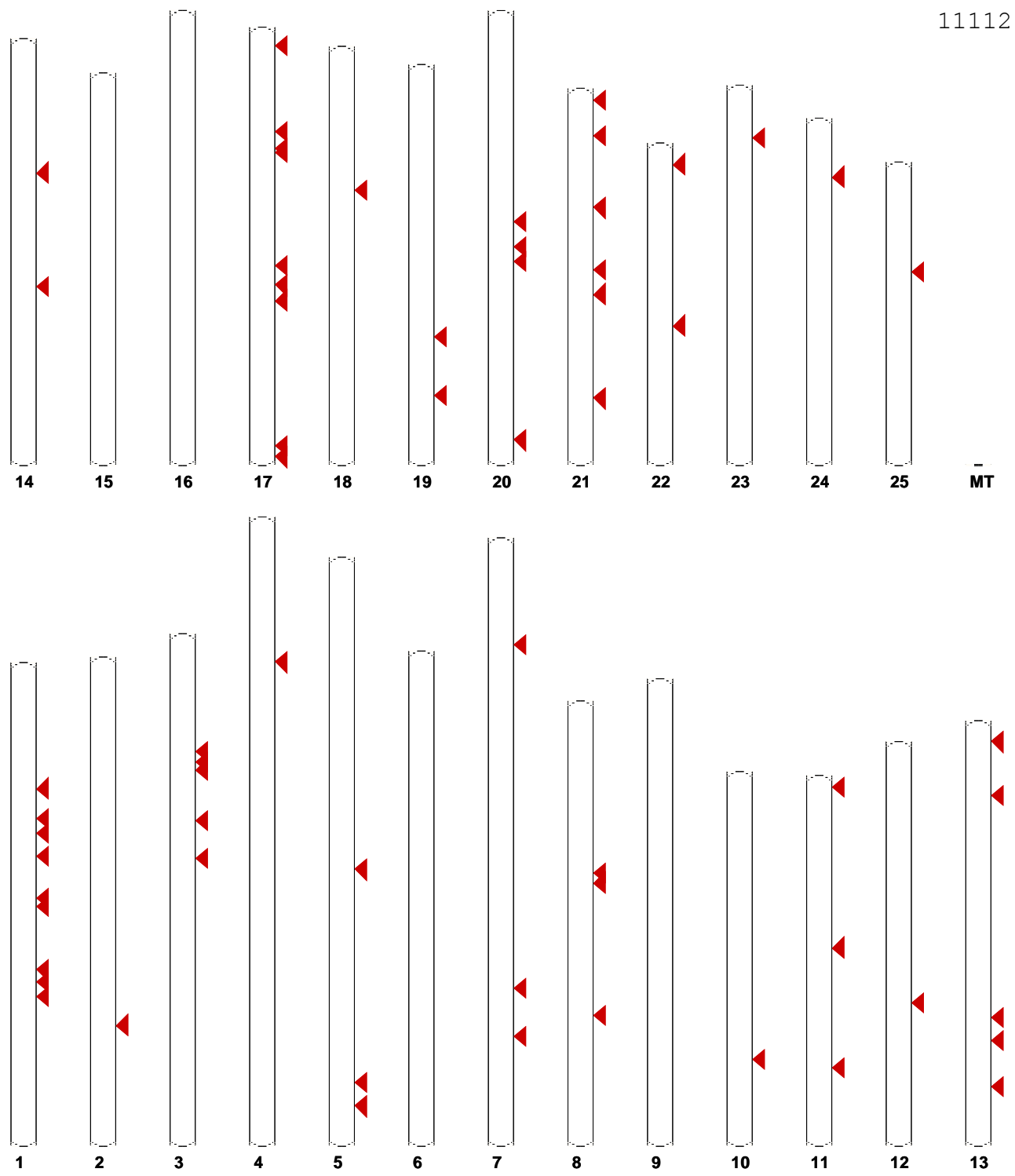

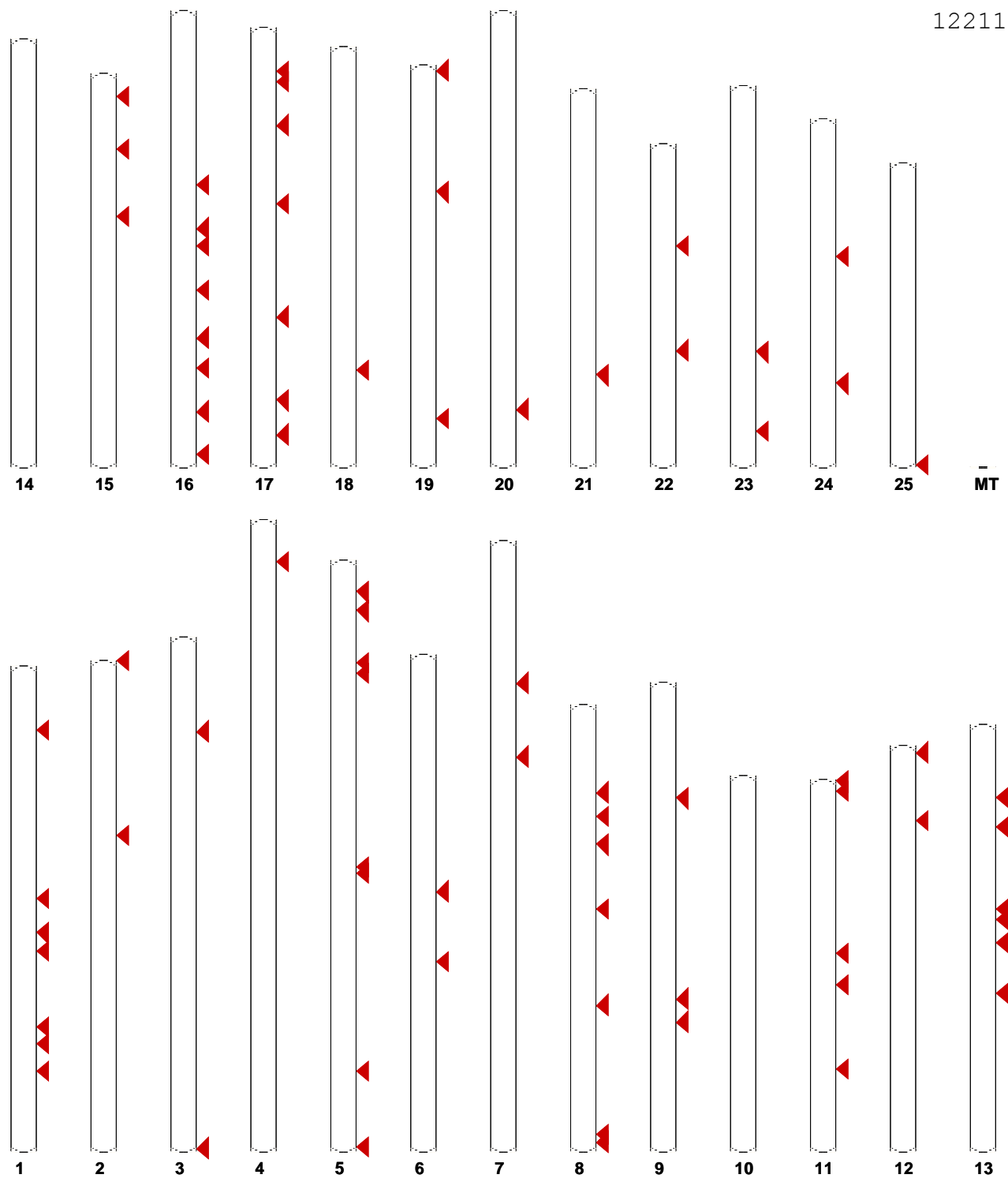

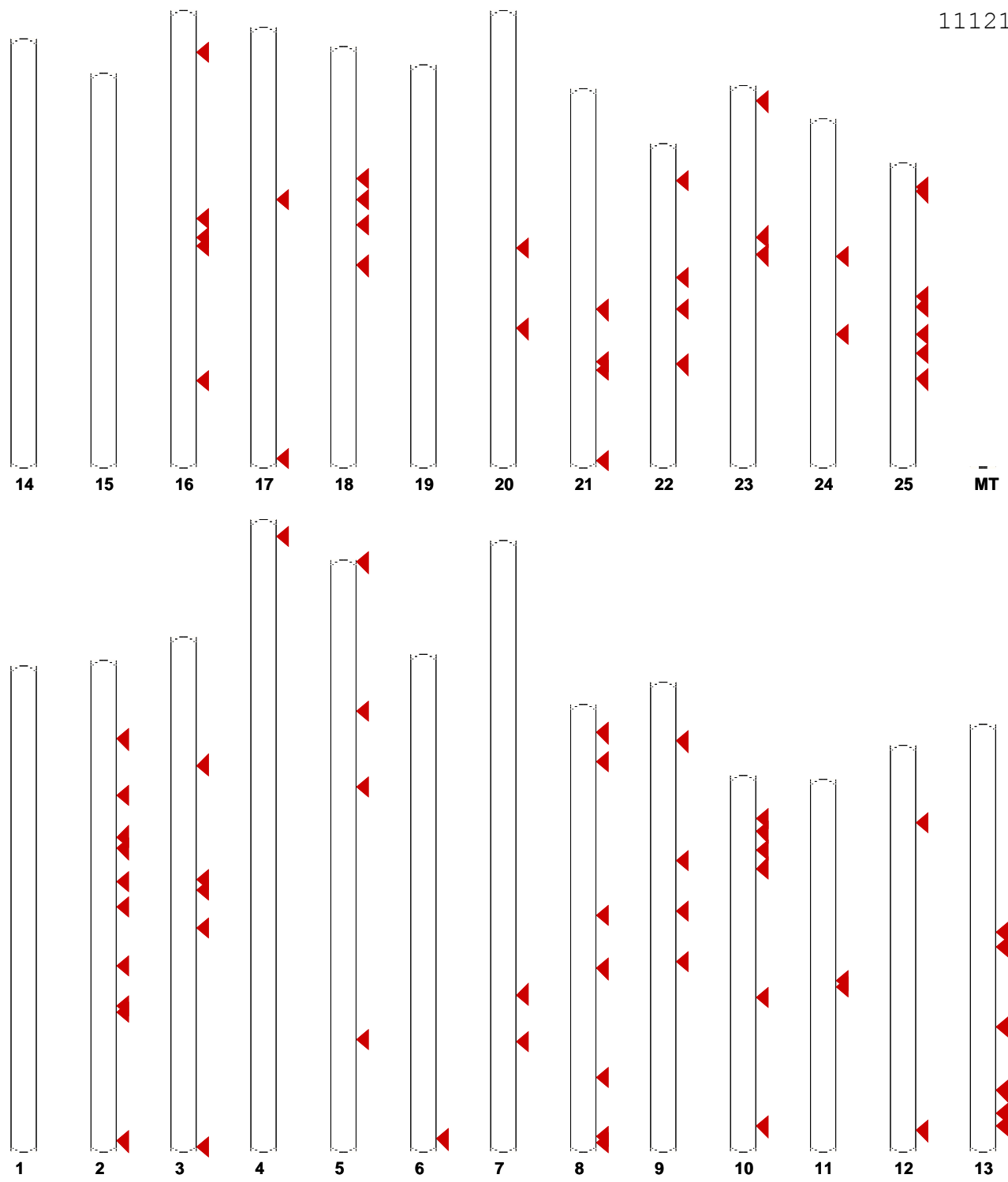

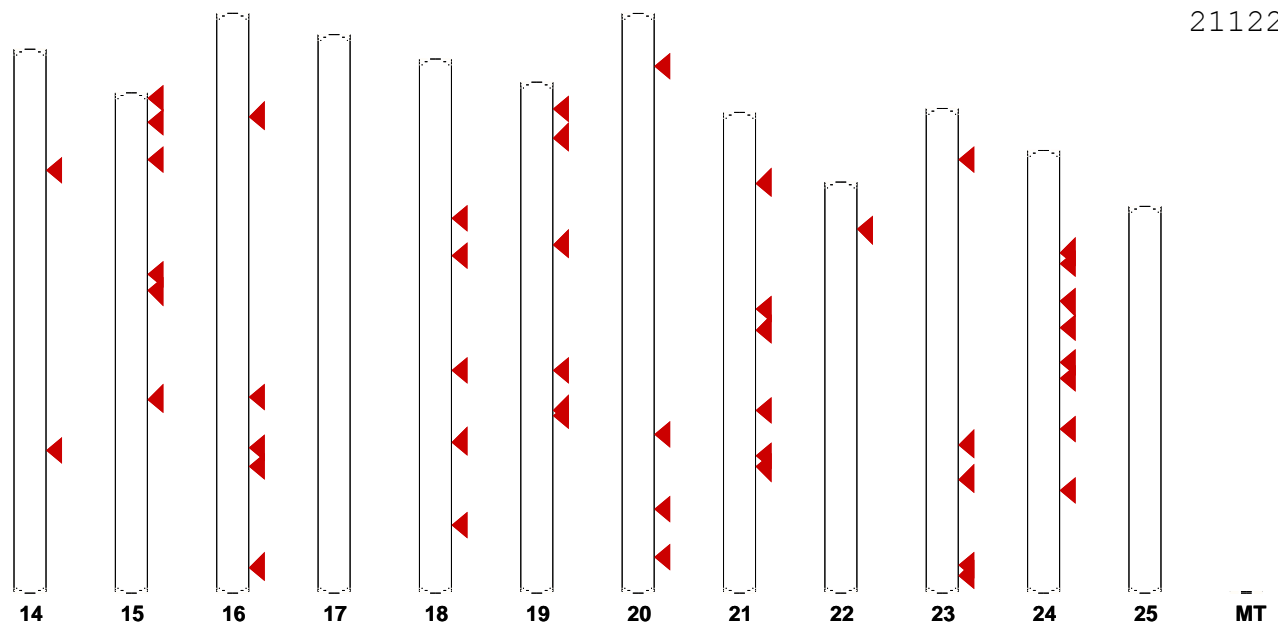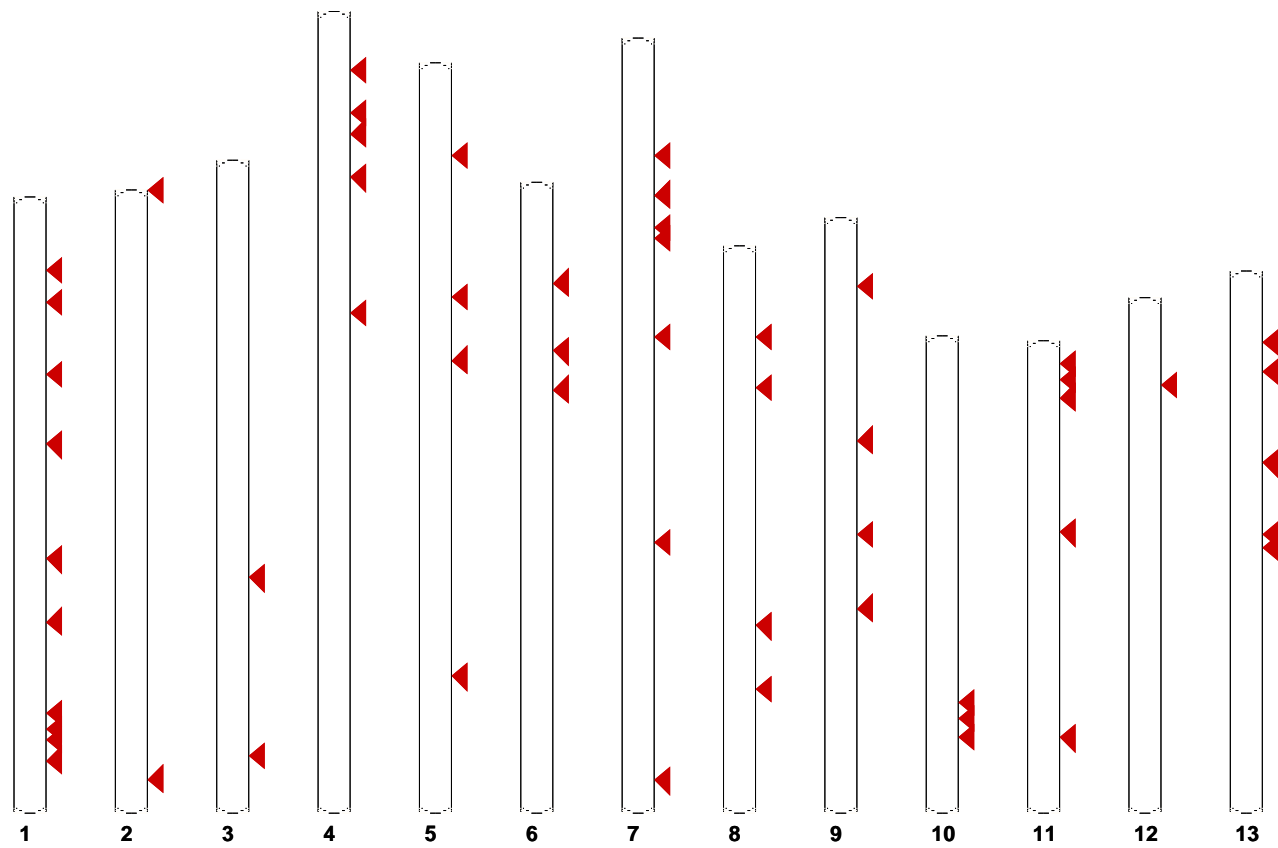

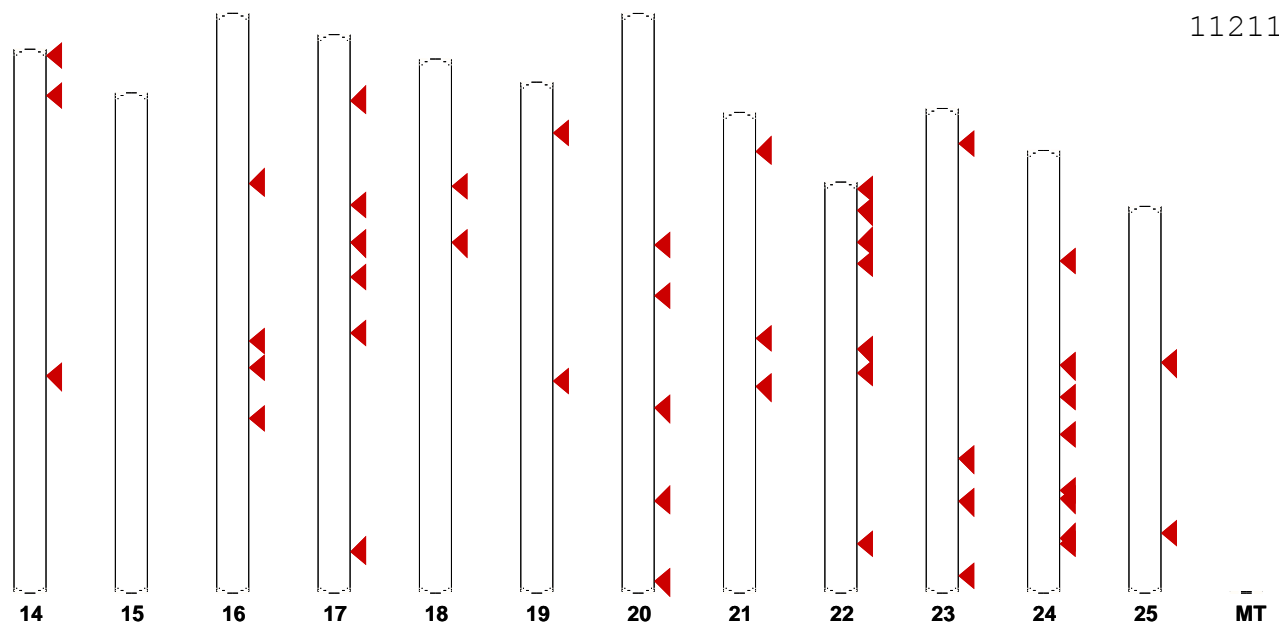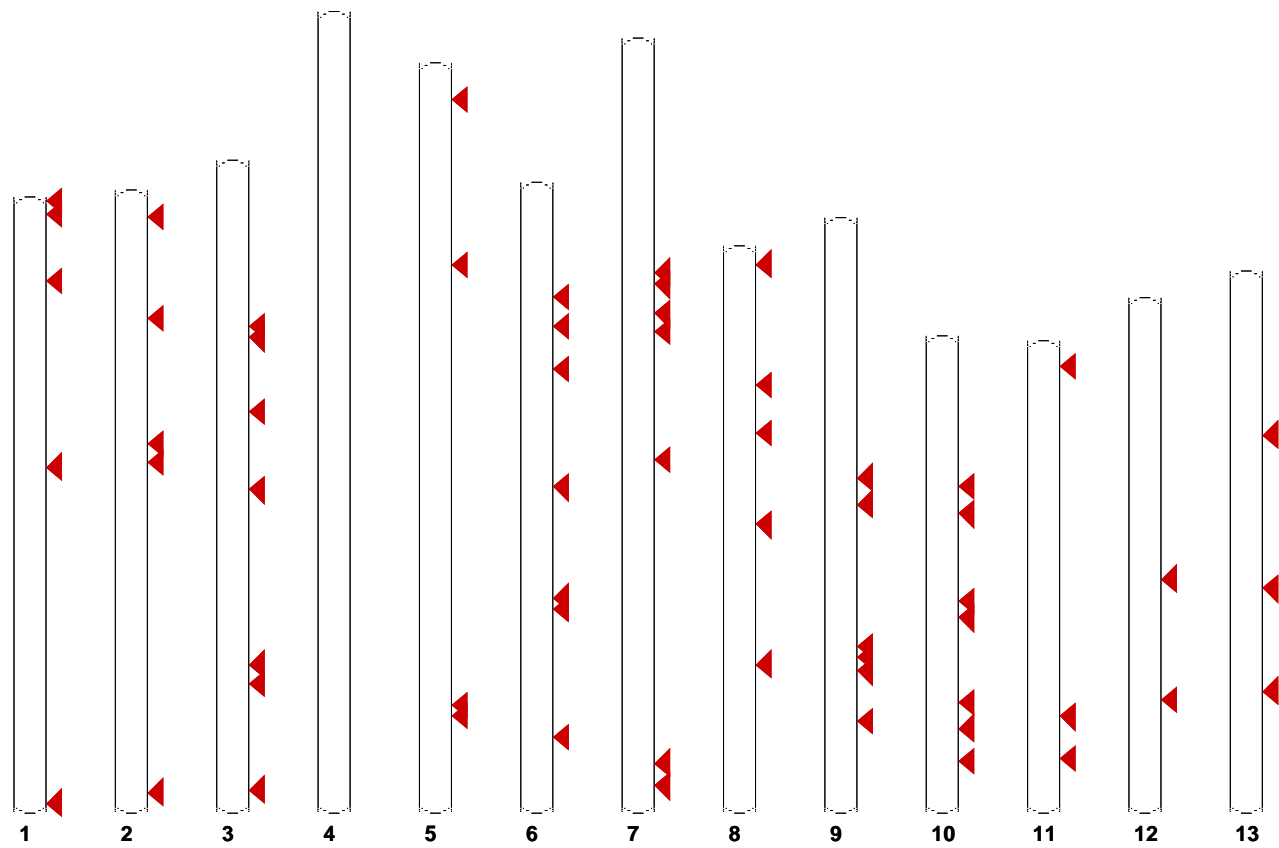

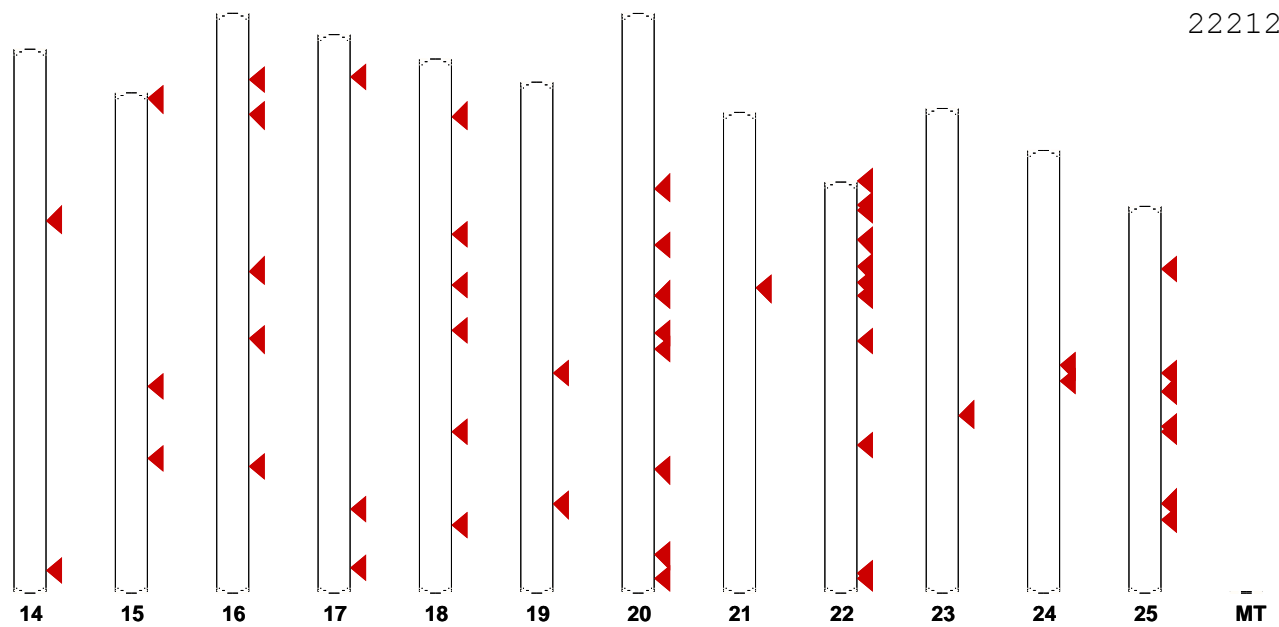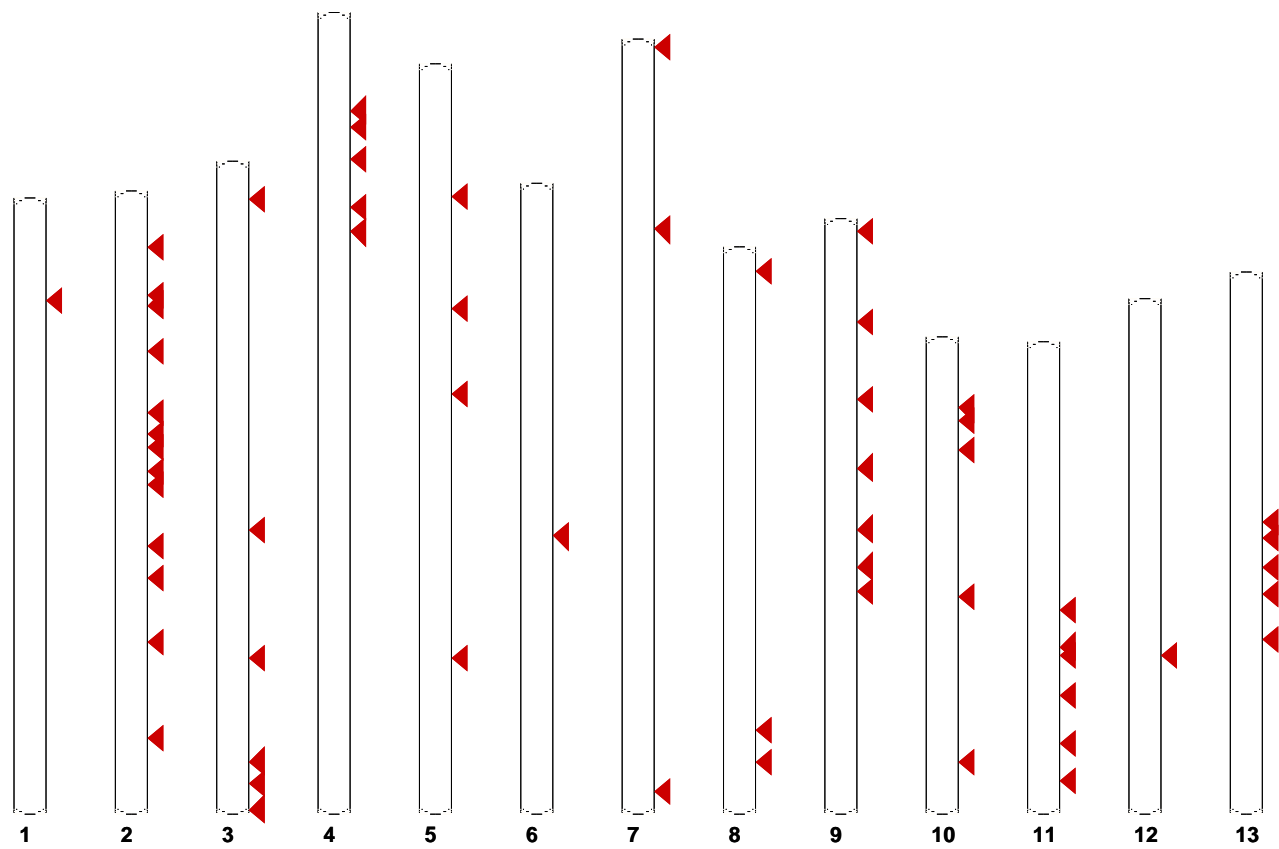

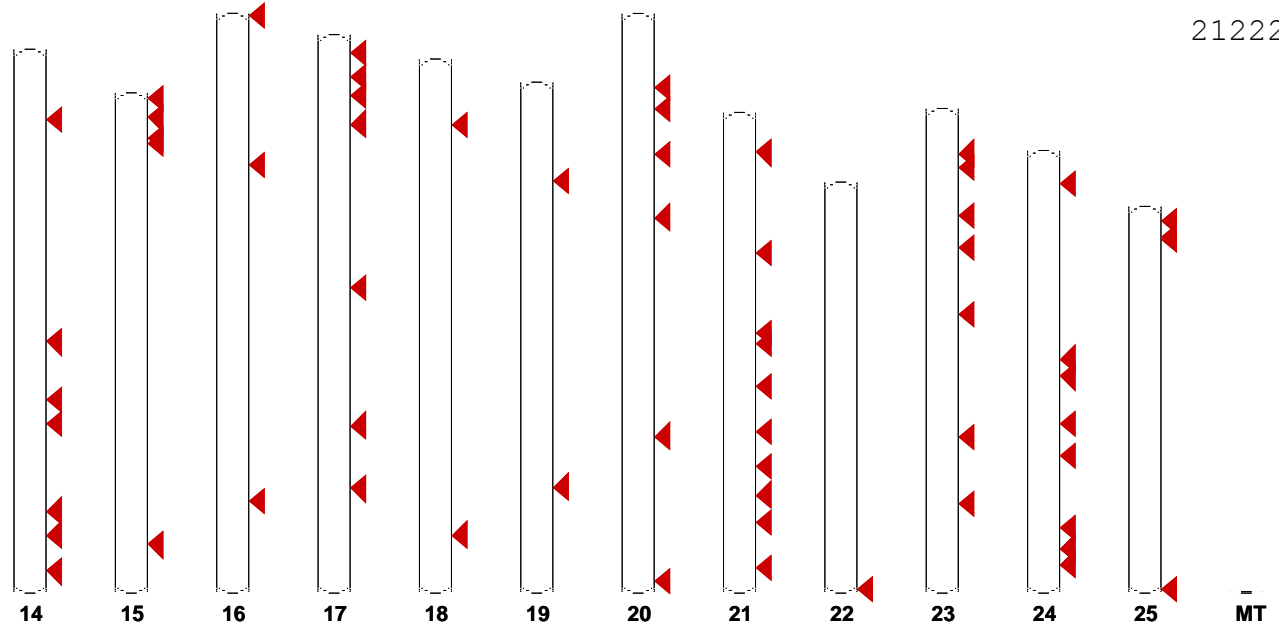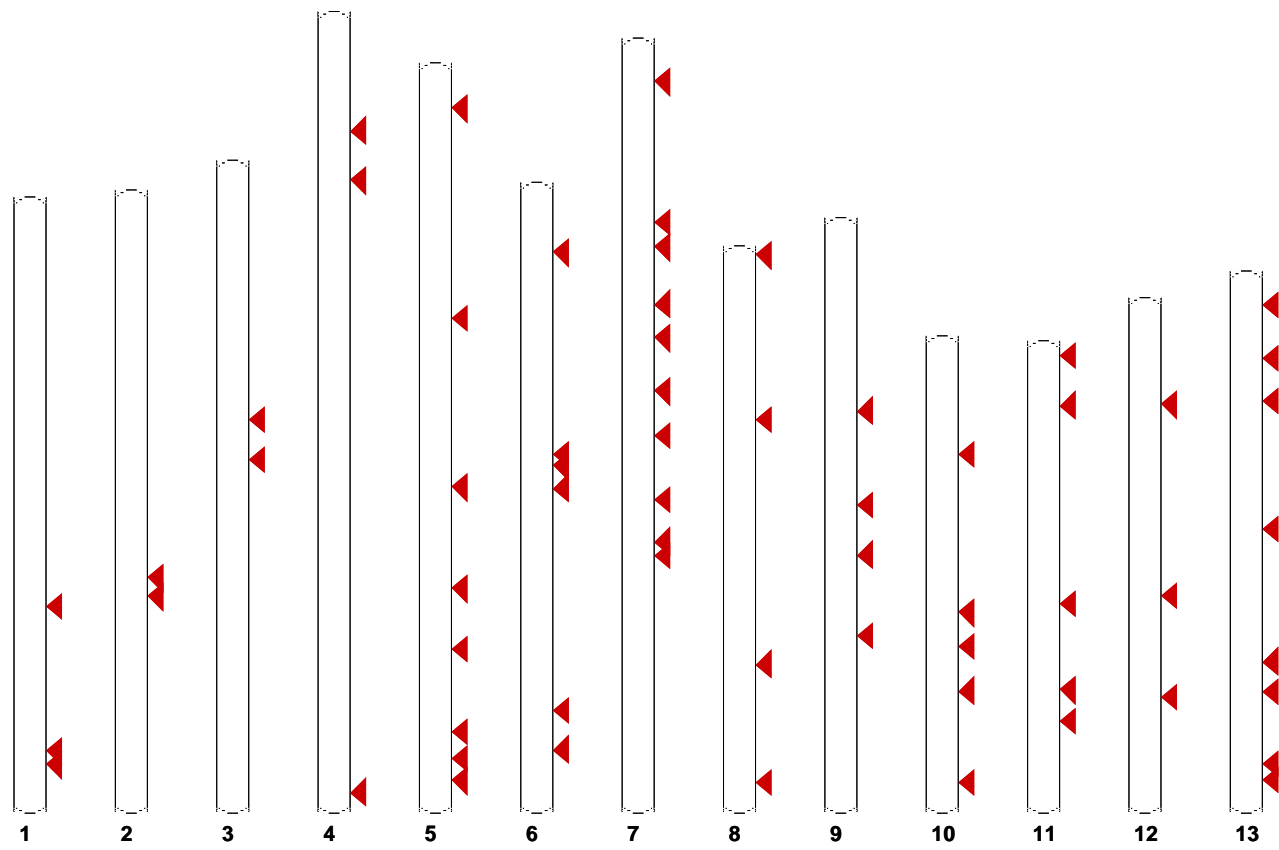

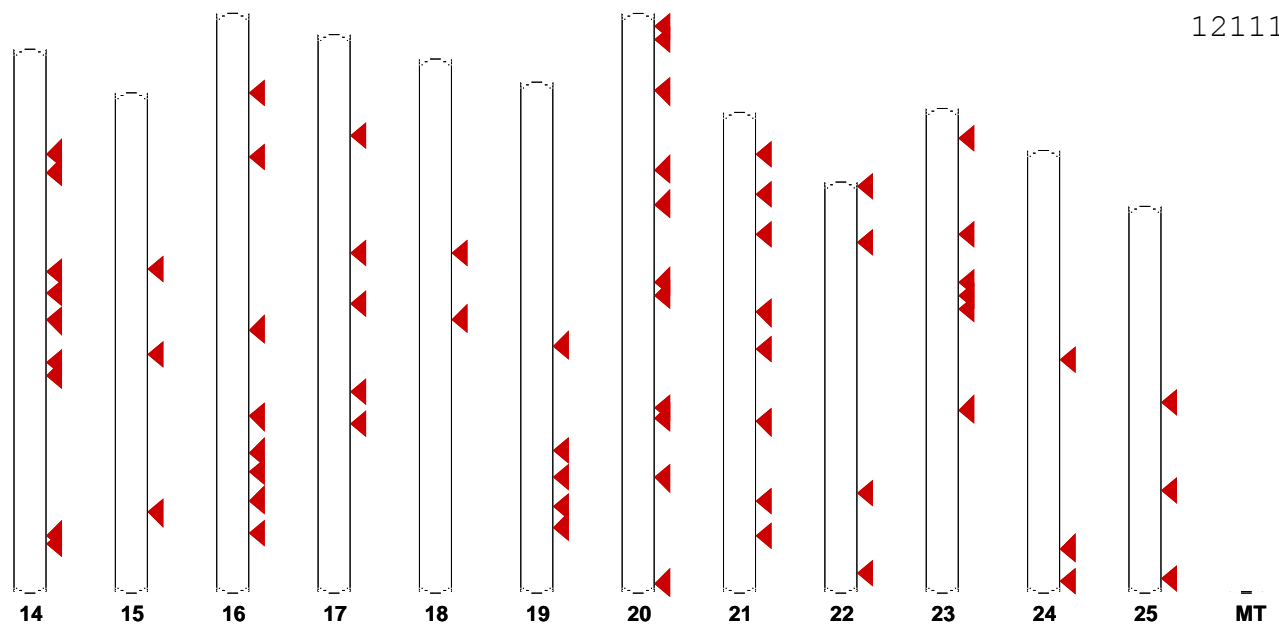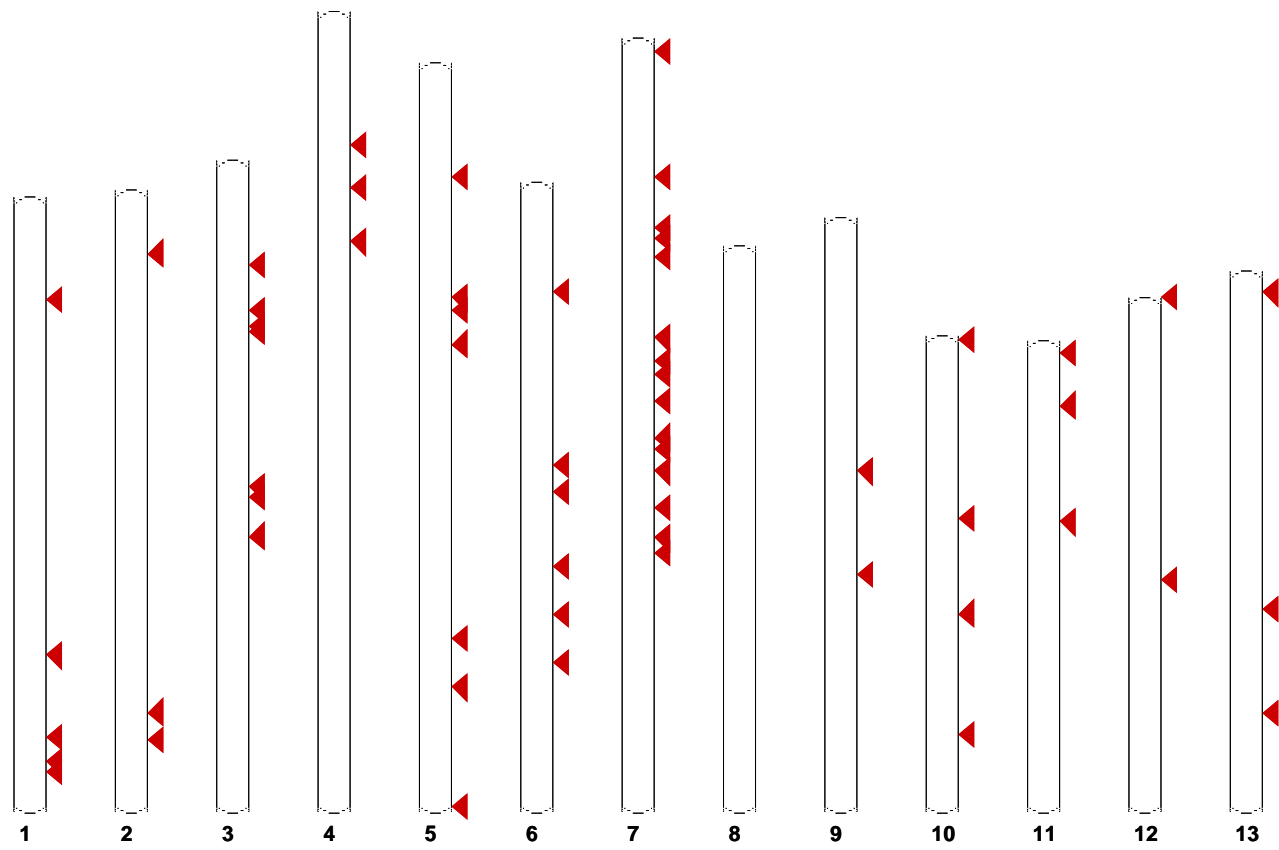

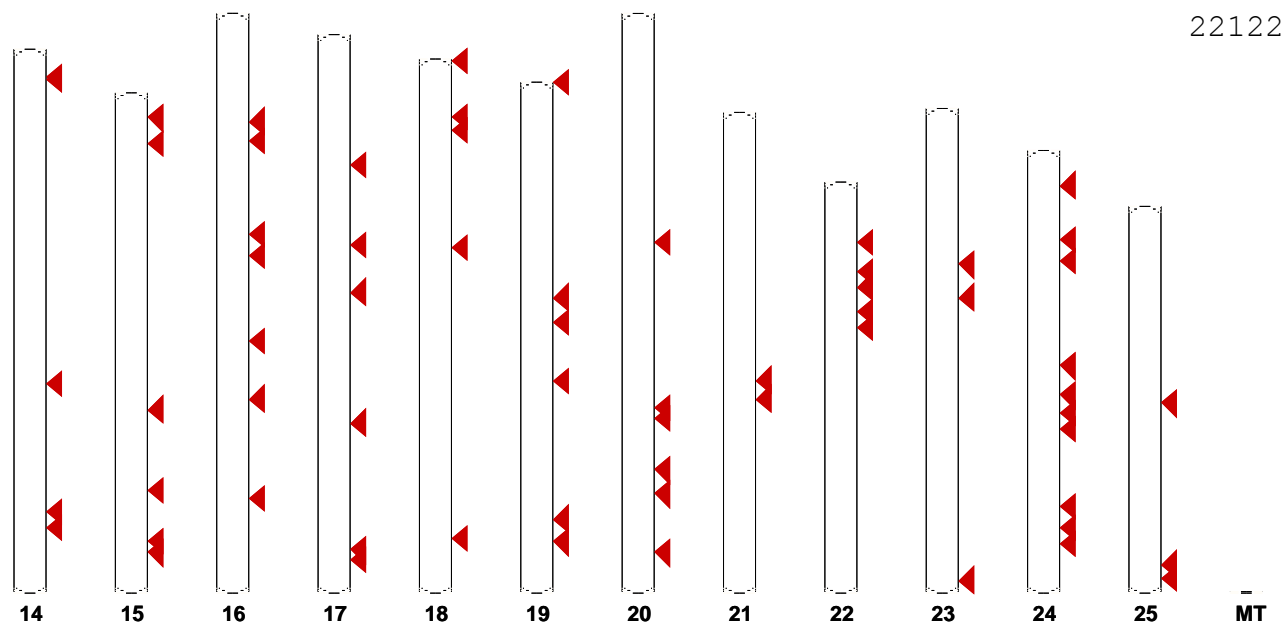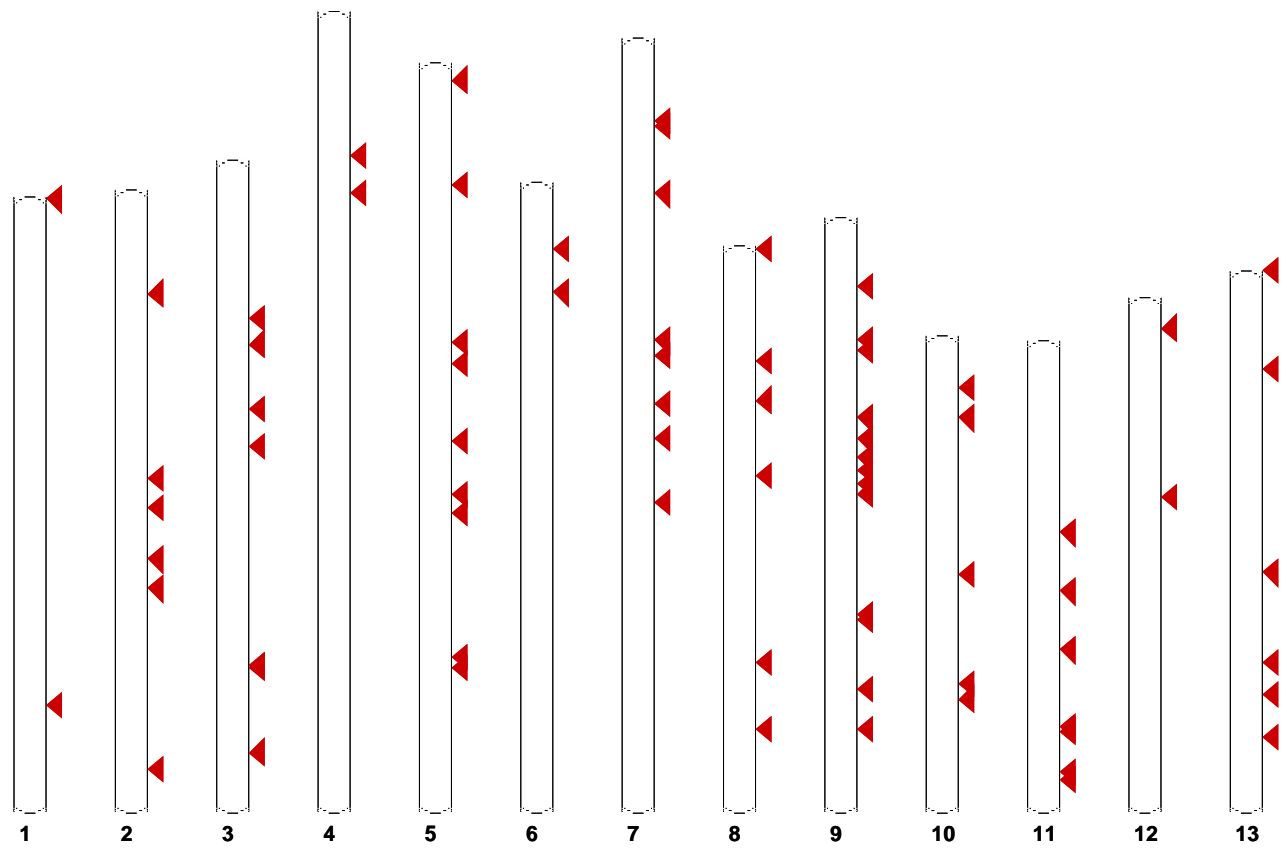

Supplement: S3 Fig — Each page shows the schematic representation of the chromosome location of genes (red arrowhead) from a mother-specific gene-expression cluster as indicated by a 5-digit code (1 = high; 2 = low) in the upper right corner of the page. (PDF) [file pone.0147151.s003.pdf]

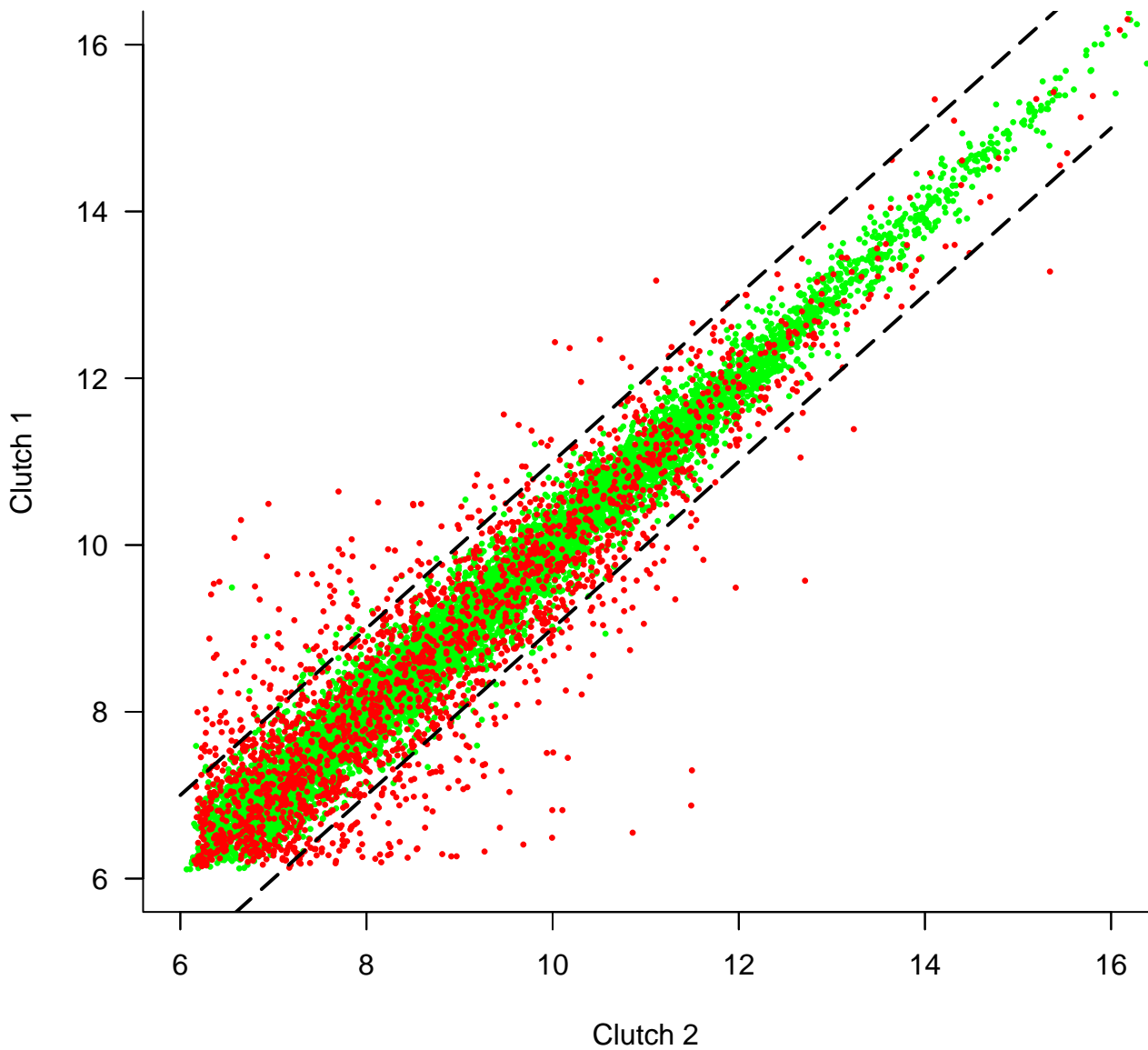

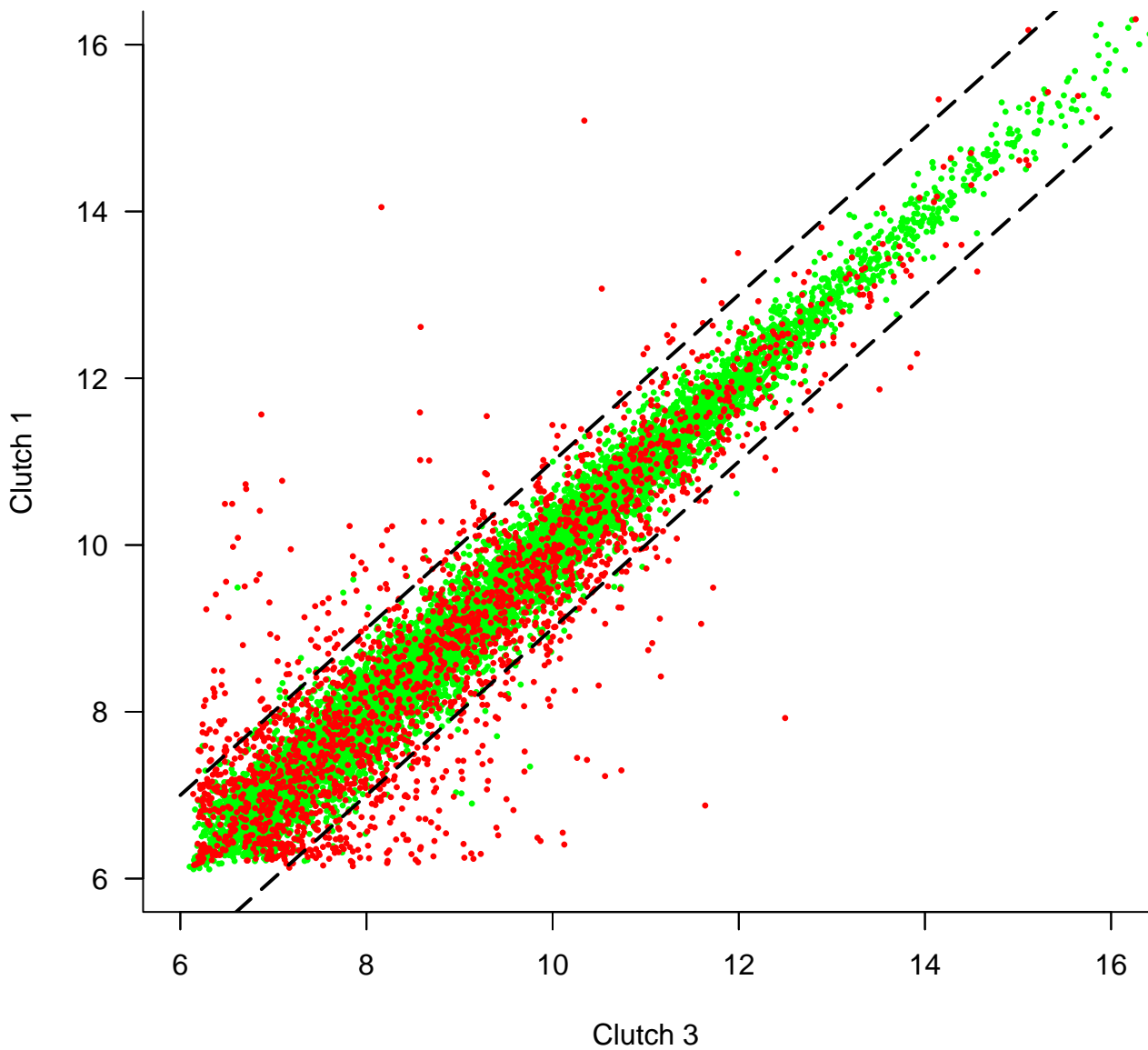

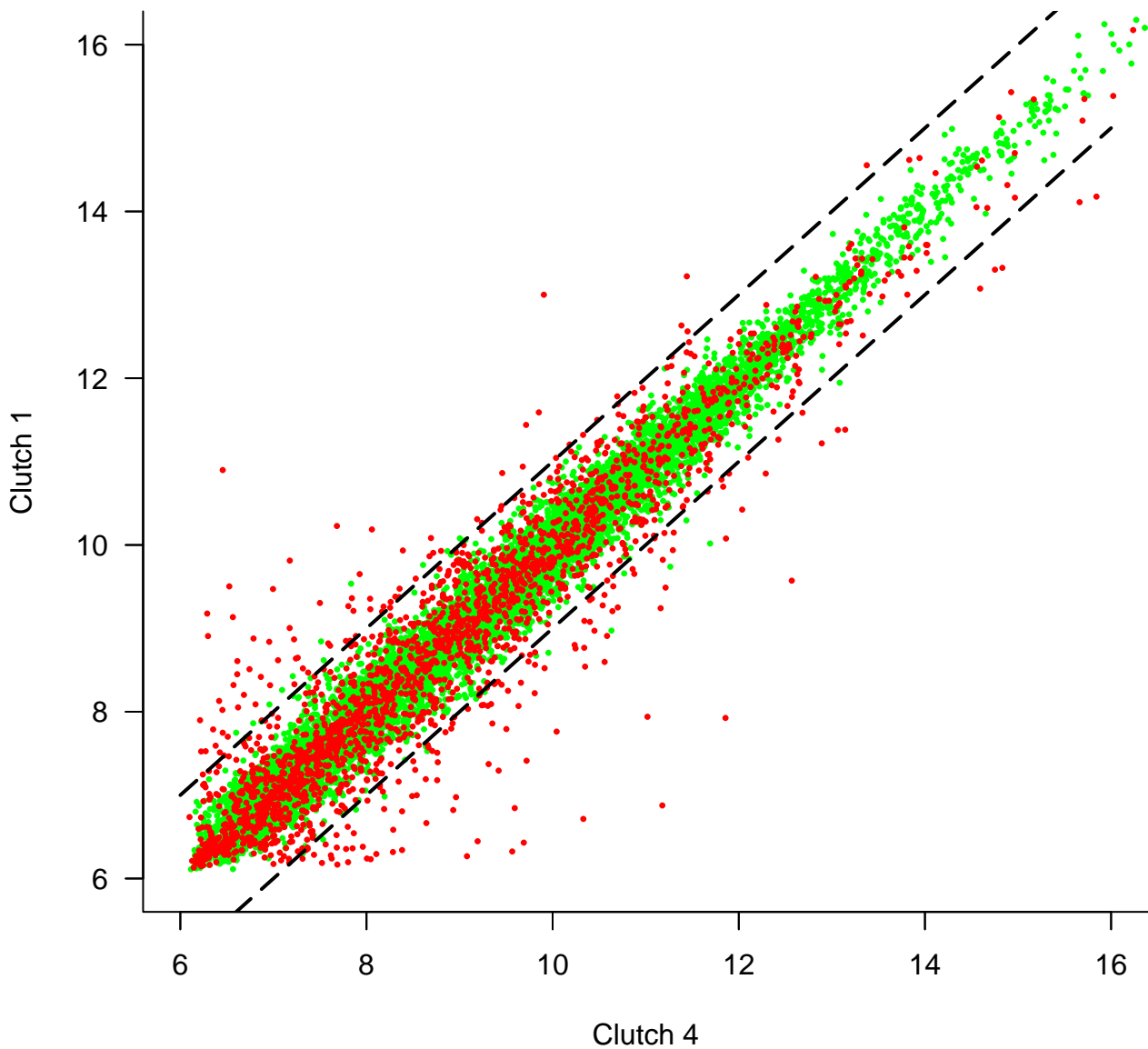

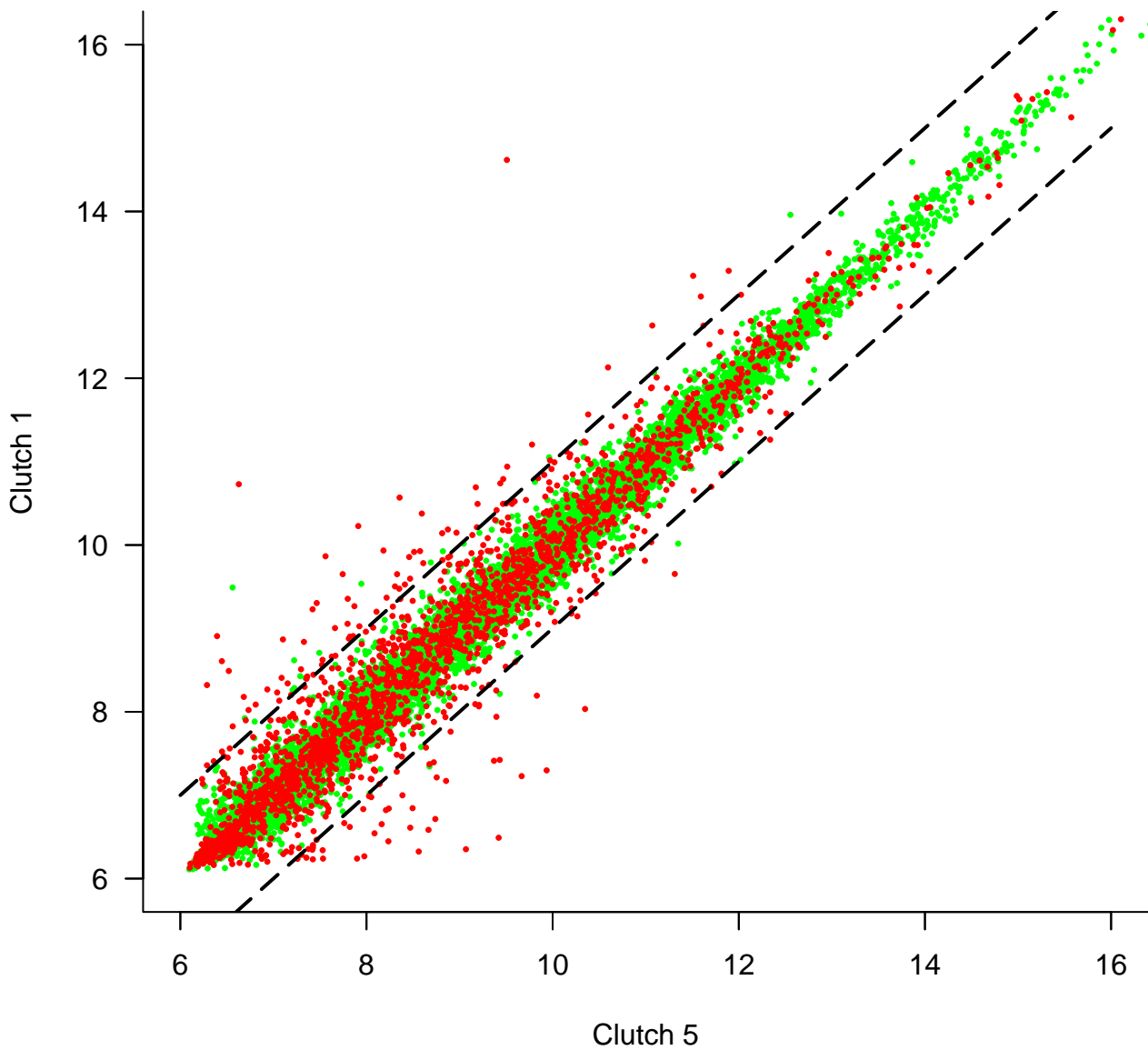

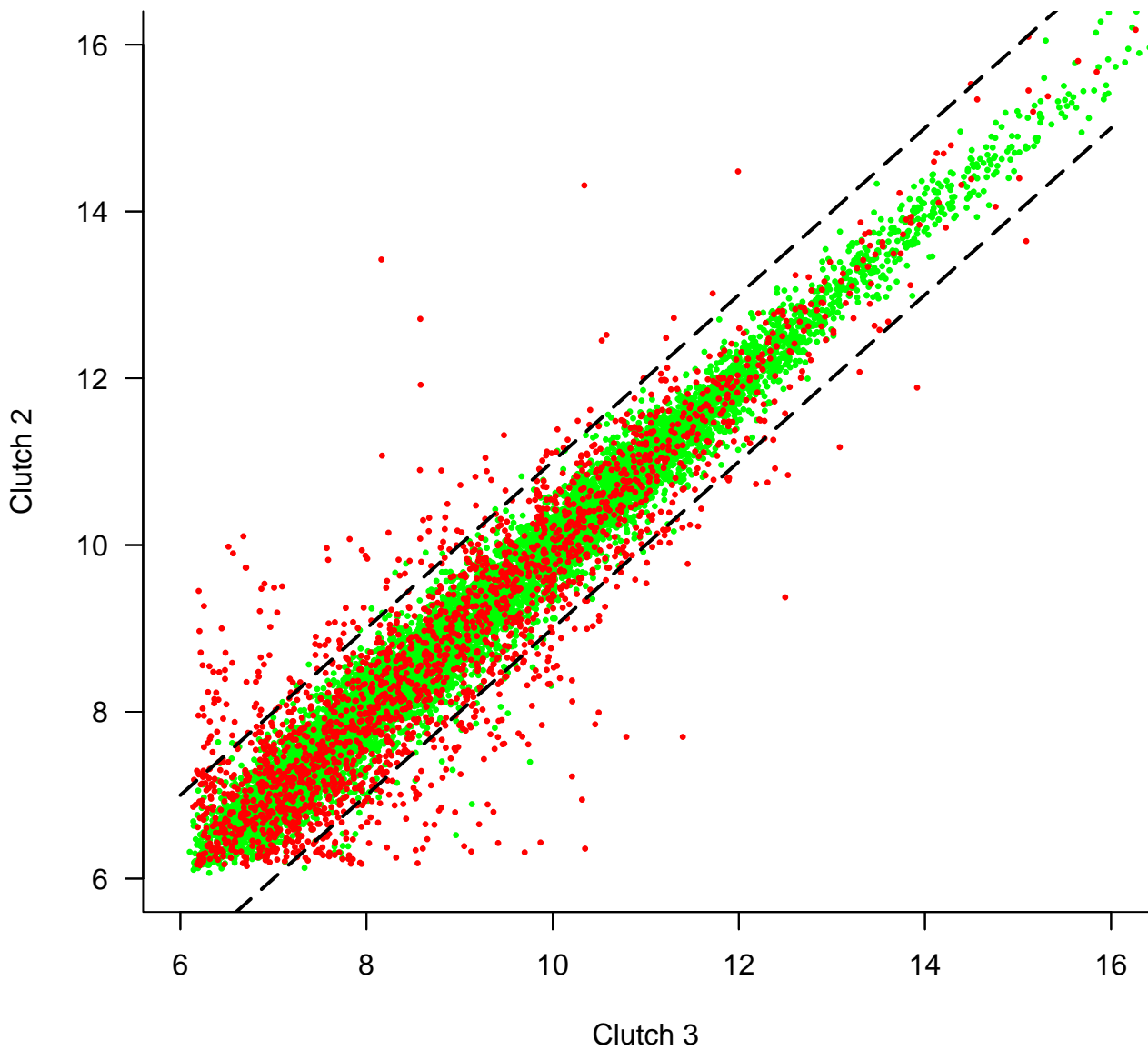

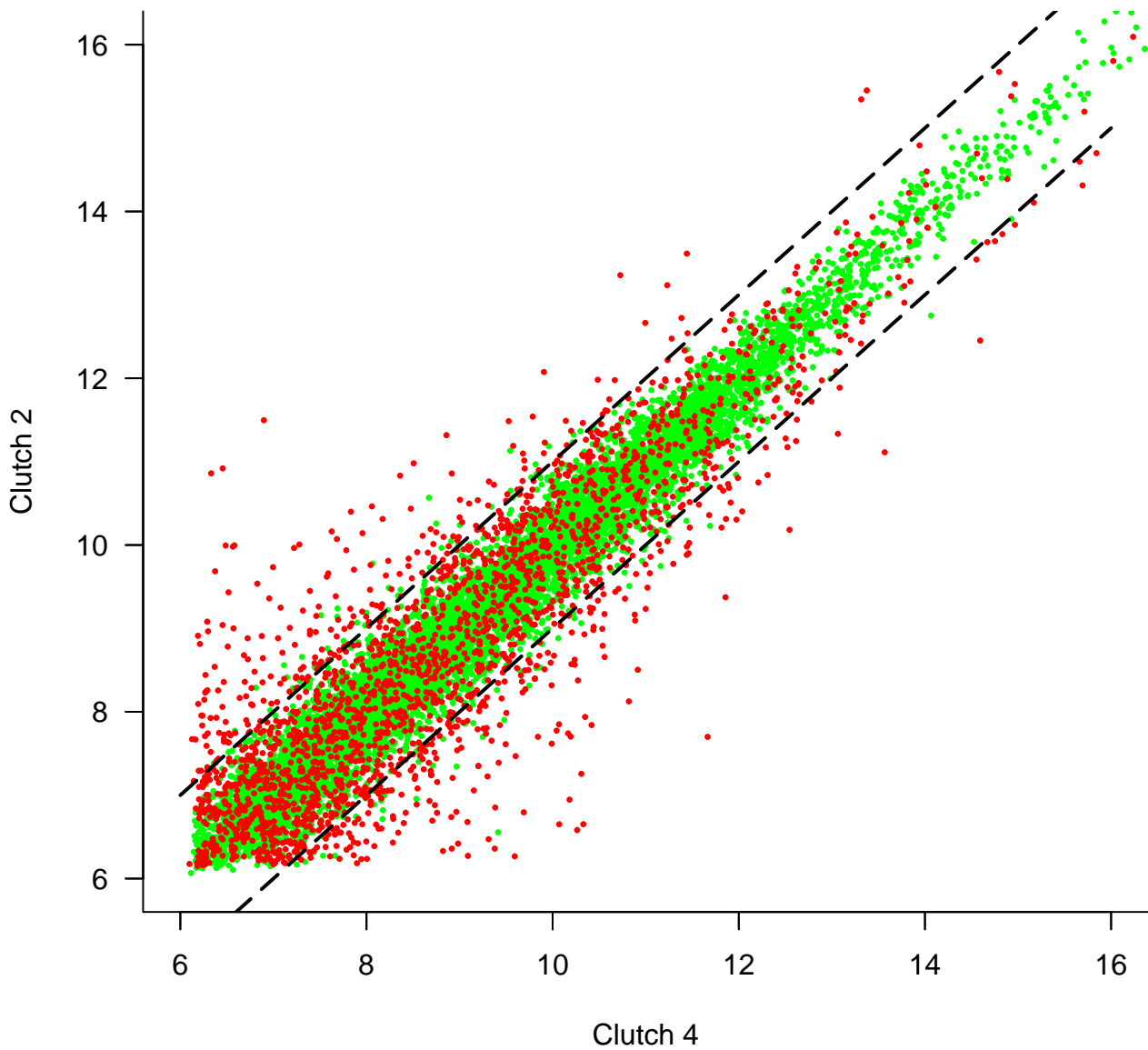

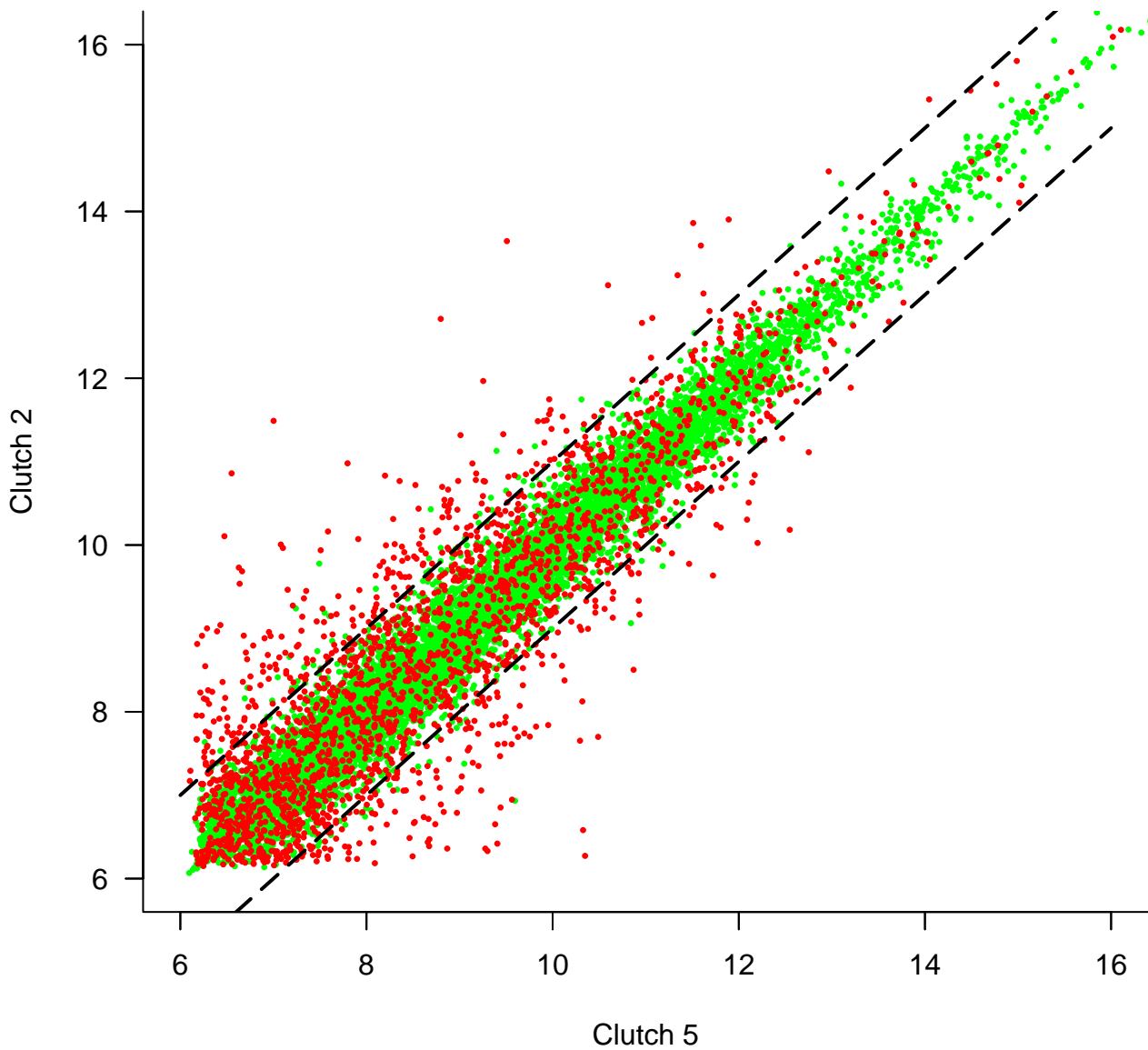

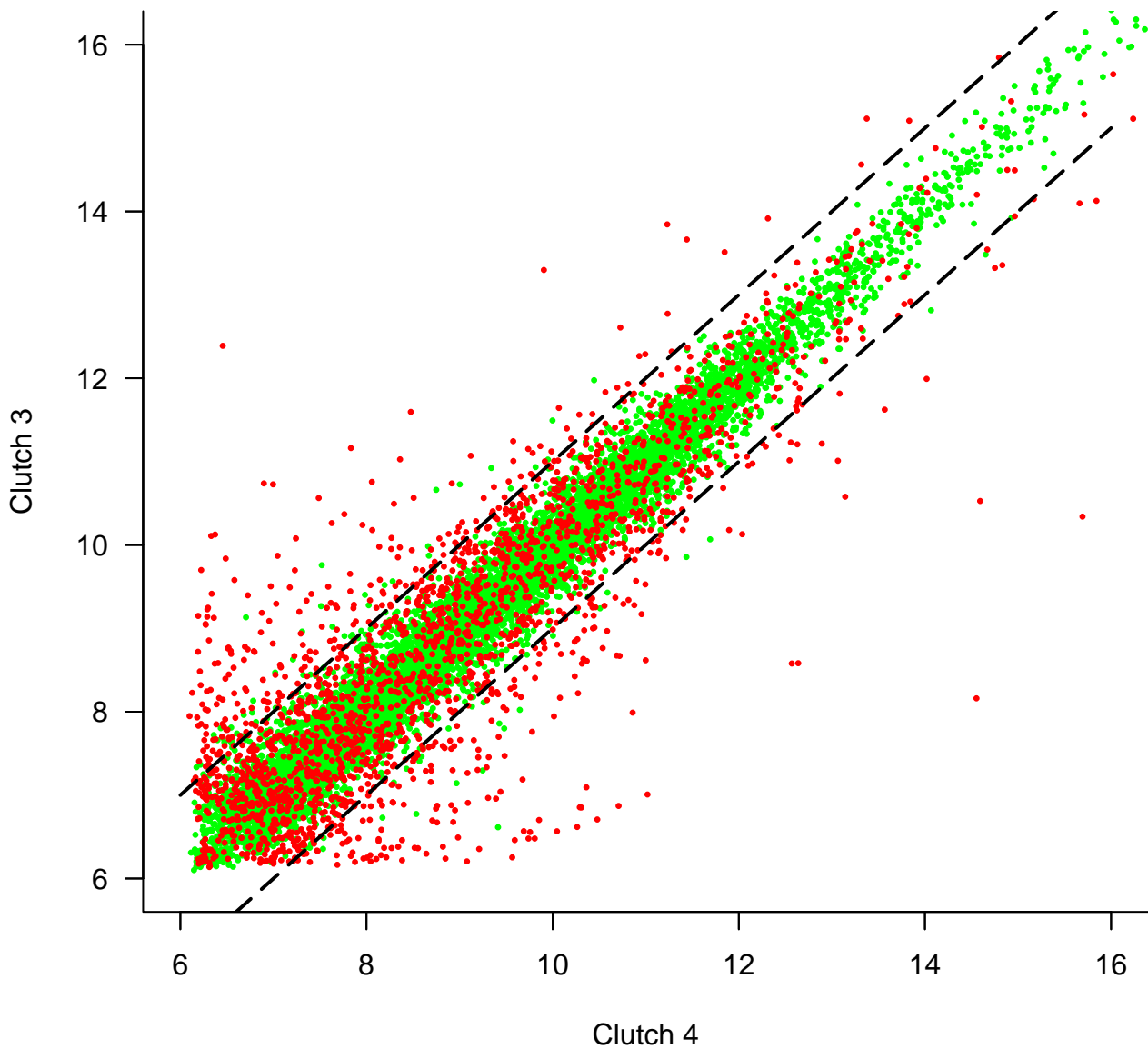

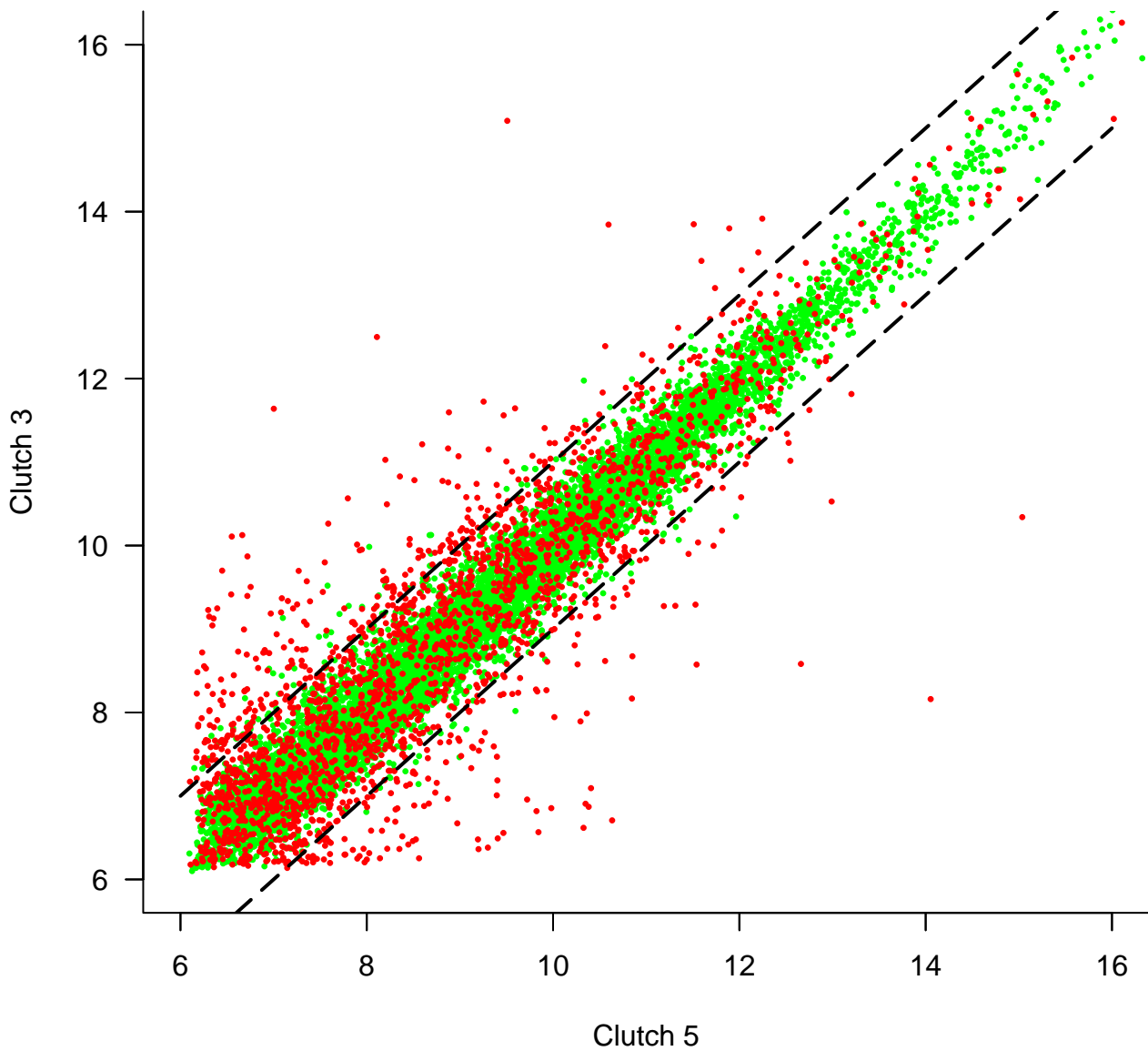

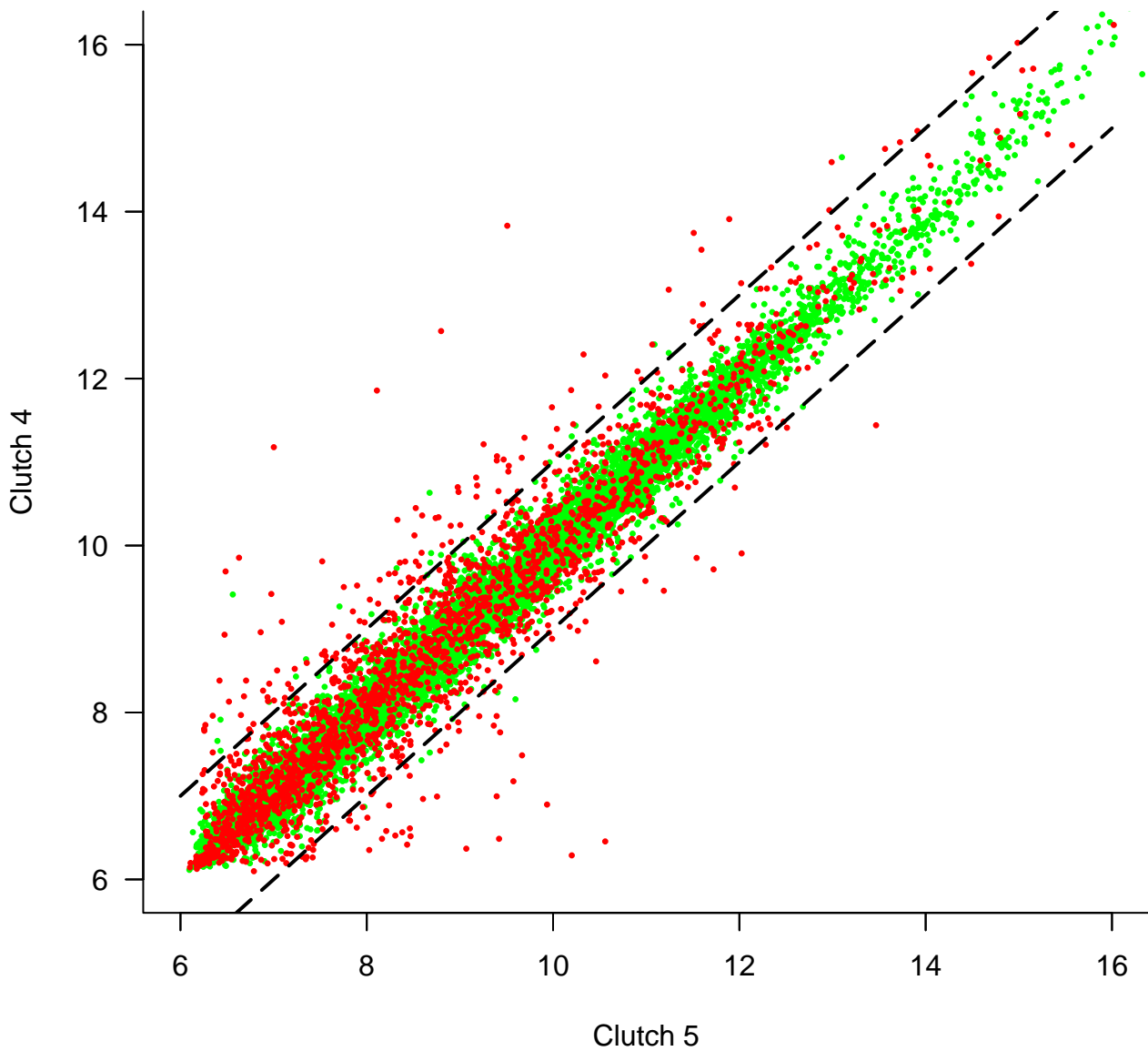

Supplement: S14 File — Red dots represent the 2,224 genes that show an absolute distinct mother-specific expression level; green dots represent all other expressed genes. Dashed lines represent a fold change of 2 between the 2 clutches. (PDF) [file pone.0147151.s017.pdf]
