# Supplementary material for: Mother-Specific Signature in the Maternal Transcriptome Composition of Mature, Unfertilized Zebrafish Eggs
Source: PLoS One. 2016 Jan 22;11(1):e0147151. doi: 10.1371/journal.pone.0147151 (PMC4723340; doi:10.1371/journal.pone.0147151)
Supplement: S3 File — (DOCX) [file pone.0147151.s006.docx]

**Ribo-depletion protocol**

**Materials**

Binding buffer (5 mM Tris-Cl pH 7.5, 1 mM EDTA, 1 M NaCl)

Dynabeads MyOne Streptavidin C1 (Thermo Fisher Scientific)

E.Z.N.A. MicroElute CleanUp Kit (Omega Biotek)

3’-biotinylated single-stranded DNA oligonucleotides (1.5 pmol/μl/oligo) (Eurogentec)

**Procedure**

1. Wash 50 µl streptavidin C1 beads per sample two times with one volume of H_2_O
2. Wash once with one volume of binding buffer
3. Resuspend washed beads in 20 µl binding buffer and keep at 37°C until use
4. Prepare the following capture mix:

2 μl total RNA (1 μg)

1 μl biotinylated oligos

30 µl binding buffer

1. Incubate for 5 min at 70°C in a thermocycler
2. Cool down to 37°C at -0.02°C/s
3. Add 20 µl washed beads
4. Incubate for 15 min at 37°C
5. Use magnetic separation to transfer the supernatant containing ribo-depleted RNA to a new 1.5 ml tube
6. Wash the beads by resuspension in 50 µl binding buffer
7. Recover supernatant by magnetic separation and combine with the first eluate
8. Remove residual beads by centrifugation at 15,500 x g for 5 min
9. Transfer supernatant to a new 1.5 ml tube
10. Adjust the volume to 100 μl with RNase-free H_2_O
11. Perform RNA cleanup using E.Z.N.A. MicroElute CleanUp Kit
12. Elute with 16 µl RNase-free water
13. Store the ribo-depleted RNA at -80°C until use
